# Empirical evidence for concerted evolution in the 18S rDNA region of the planktonic diatom genus *Chaetoceros*

**DOI:** 10.1038/s41598-020-80829-6

**Published:** 2021-01-12

**Authors:** Daniele De Luca, Wiebe H. C. F. Kooistra, Diana Sarno, Elio Biffali, Roberta Piredda

**Affiliations:** 1 Department of Integrative Marine Ecology, Stazione Zoologica Anton Dohrn, Villa Comunale, 80121 Naples, Italy; 2 Department of Research Infrastructure for Marine Biological Resources, Stazione Zoologica Anton Dohrn, Villa Comunale, 80121 Naples, Italy; 3 Present Address: Department of Biology, University of Naples Federico II, Botanical Garden of Naples, Via Foria 223, 80139 Naples, Italy

**Keywords:** Molecular evolution, Haplotypes, Evolutionary biology

## Abstract

Concerted evolution is a process of homogenisation of repetitive sequences within a genome through unequal crossing over and gene conversion. This homogenisation is never fully achieved because mutations always create new variants. Classically, concerted evolution has been detected as “noise” in electropherograms and these variants have been characterised through cloning and sequencing of subsamples of amplified products. However, this approach limits the number of detectable variants and provides no information about the abundance of each variant. In this study, we investigated concerted evolution by using environmental time-series metabarcoding data, single strain high-throughput sequencing (HTS) and a collection of Sanger reference barcode sequences. We used six species of the marine planktonic diatom genus *Chaetoceros* as study system. Abundance plots obtained from environmental metabarcoding and single strain HTS showed the presence of a haplotype far more abundant than all the others (the “dominant” haplotype) and identical to the reference sequences of that species obtained with Sanger sequencing. This distribution fitted best with Zipf’s law among the rank abundance/ dominance models tested. Furthermore, in each strain 99% of reads showed a similarity of 99% with the dominant haplotype, confirming the efficiency of the homogenisation mechanism of concerted evolution. We also demonstrated that minor haplotypes found in the environmental samples are not only technical artefacts, but mostly intragenomic variation generated by incomplete homogenisation. Finally, we showed that concerted evolution can be visualised inferring phylogenetic networks from environmental data. In conclusion, our study provides an important contribution to the understanding of concerted evolution and to the interpretation of DNA barcoding and metabarcoding data based on multigene family markers.

## Introduction

The first DNA hybridisation studies conducted in the mid-1960–1970s showed that a large fraction of eukaryotic genomes was composed of repetitive regions^1,2^. When comparing the repetitive DNA families, a greater sequence similarity within species than between them was observed^3,4^. Such observation was incompatible with the common model of divergent evolution, according to which the differences in nucleotide sequence between different repeats of the same species were expected to be as large as those between repeats of different species^5^. Therefore, there had to be a mechanism responsible for the homogenisation of such sequences. The expression “concerted evolution”^6^ was coined to indicate this phenomenon, by which an individual member of a gene family evolves in the same (concerted) way as all the other members of the family^7^.

The best-known example of concerted evolution are the ribosomal DNA cistrons (rDNA)^8,9^, but also other non-coding regions and genes (e.g. globins, immunoglobulins, heat-shock genes, histones) are known to evolve in this way^8,10–12^. Two processes, gene conversion and unequal crossing-over, will eventually lead to sequence homogeneity in absence of mutations^13–15^. However, mutations always occur and gene conversion and unequal crossing-over also contribute to their spread, generating variation across repeats^5^. These deviations from sequence homogenisation have been detected in animals^16,17^, fungi^18^, protists^19^ and especially in plants^20–24^. The extent of such deviations is important in the case of the rDNA cistron, since it is the target for DNA barcoding studies in such taxa as fungi and protists^25–27^. Therefore, understanding the inheritance of rDNA genes and spacers is vital for taxonomic and systematic studies involving them.

So far, intra-individual variation among the rDNA-cistrons has been spotted as noise in electropherograms and as variation among sequenced clones generated from the PCR products^28,29^. The resulting sequences, together with ones from closely related species, were then analysed phylogenetically to ascertain the degree of relatedness within and among species^22,30–32^. However, this approach has two main limitations: the number of detectable variants is constrained by the number of sequenced clones and there is no information about the abundance of each variant. In recent times, concerted evolution has also been revealed by whole-genome shotgun sequence data^9^ and chromosomal and array approaches^33,34^. Nowadays, the use of the high-throughput sequencing approach, which allows sequencing of thousands of copies of a target region from environmental samples and bulk communities (metabarcoding) and even single specimens (single strain HTS), could overcome the limitations associated with cloning and complexity of analysis of whole-genome shotgun data^35,36^. Therefore, metabarcoding can be particularly useful to study concerted evolution. A recent work by^37^ assessed the diversity in the marine planktonic diatom family Chaetocerotaceae through a 18S-V4 rDNA environmental metabarcode haplotype collection generated from 48 seasonal samples taken at the long-term ecological research station (LTER) MareChiara (MC) in the Gulf of Naples, Mediterranean Sea, Italy. A phylogeny inferred from the chaetocerotacean metabarcode haplotypes and (Sanger-sequenced) taxonomic references showed the following recurrent patterns. Terminal clades comprised from just a few to several hundreds of haplotypes, one of which was far more abundant than the others. That one, called “dominant haplotype”, was identical or nearly identical to the Sanger-sequence of a reference strain, when available, of that clade^37^. Furthermore, the relationship among “dominant” and “minor” haplotypes was consistent across species: when plotted on a logarithmic scale, the reads of the dominant haplotype were at least one order more abundant than those of any of other haplotypes of that species. Based on that signal, the authors hypothesized that such a pattern “result from an equilibrium between the appearance of novel haplotypes, random drift, and the homogenizing effect of concerted evolution”^37^.

In the present study, we designed an experiment and generated new data to confirm the hypothesis of concerted evolution in the 18S rDNA gene of several *Chaetoceros* species by high-throughput sequencing (HTS) of the V4 region of monoclonal strains. In particular, we used the new data obtained from monoclonal strains together with the previous environmental chaetocerotacean metabarcode dataset and the *Chaetoceros* reference barcodes obtained with Sanger sequencing to test if: (i) the most abundant haplotype in each single strain matches the reference barcode of that strain obtained with Sanger sequencing and with the dominant environmental haplotype; (ii) the minor haplotypes found in the environmental metabarcoding data are present in the strains of each species; (iii) the temporal distribution of dominant and minor haplotypes in environmental data is the same.

## Methods

### Selection of taxa to study concerted evolution

In order to test the aforementioned hypotheses, we used the metabarcoding data of Chaetocerotaceae from the LTER MareChiara (Gulf of Naples) from^37^ deposited in GenBank at the accession numbers MK938374–MK940235 (414,041 reads). The dataset includes sequences gathered from 48 dates across three years (2011–2013); sampling procedure is described in detail in^38^. In this study, the term “haplotype” indicates the non-redundant (unique) sequences. Based on the chaetocerotacean metabarcode haplotype diversity illustrated in the phylogeny presented in^37^, we selected for detailed HTS analysis strains of six species representing different clades of the family tree and showing different read distribution patterns over the environmental haplotypes or over the seasonal cycle. In particular, we chose: *Chaetoceros tenuissimus* as representative of species occurring at high abundance all over the year and displaying many minor haplotypes; *C. costatus* as species with a marked seasonality, displaying also a few minor haplotypes at high abundances; *C. anastomosans* as a relatively rare species, displaying a single, lowly abundant, dominant haplotype in the environmental data; *C. curvisetus* 2 as species common all over the year with few minor haplotypes; *Chaetoceros* spp. Na11C3 and Na26B1 as examples of two closely related species, i.e., with distinct reference barcodes and dominant haplotypes, but recovered together in an internally unresolved clade with minor haplotypes. For each species, we selected outgroup taxa (Supplementary Table S1) for subsequent validation of sequences gathered from BLAST analysis.

### Analysis of environmental sequences

As time-series data we used V4 metabarcode reads generated from 48 plankton samples taken at the Long Term Ecological Research (LTER) station MareChiara in the Gulf of Naples (Mediterranean Sea) over the seasonal cycles of three consecutive years (2011–2013)^37,38^. To retrieve sequences of the selected *Chaetoceros* species in the chaetocerotacean dataset, we used the respective 18S reference sequences and close outgroups as queries for a local BLAST^39^ at 95%. The metabarcodes extracted were then aligned with the references and the outgroup taxa using MAFFT online^40^ and a phylogenetic tree was built in FastTree v2.1.8^41^, using the GTR model. The resulting tree was visualised and modified in Archaeopteryx v0.9901^42^ in order to remove sequences clustering within outgroup clades and gather only metabarcodes of the species of interest. The sequences retrieved were considered validated and used to retrieve the info of abundance using mothur v1.41.1^43^.

### Single strain HTS

Single strain metabarcoding was performed on: two strains of *C. anastomosans*, four strains of *C. costatus*, four strains of *C. curvisetus* 2, one of *Chaetoceros* sp. Na26B1, two of *Chaetoceros* sp. Na11C3 and three strains of *C. tenuissimus* (Table 1). Strains were obtained from cell chains collected at the LTER MareChiara. For each sample, we performed individual PCR in two steps: a first reaction for the amplification of the target sequence, and a second reaction (using the PCR product of the former one as template) to ligate proprietary adaptor sequence (P1) and unique 10–12 bp long identifier nucleotide key tags (barcodes) compatible with the GeneStudio S5 Ion Torrent (Life Technologies, Carlsbad, California). The obtained fragment contained all the information required for sequencing and differentiation (barcoding) of samples. A detailed description of the procedure is provided in Supplementary File S1.

**Table 1:** List of strains utilised for single-strain HTS.

Species	Strain
*C. anastomosans*	Na14C2
	Na14C3
*C. costatus*	Na1A3
	Na32B1
	Ro1B1
	Ro2A2
*C. curvisetus* 2	Ch5B2
	Na1C1
	Na19A2
	Na20A4
*Chaetoceros* sp. Na11C3	Na11C3
	Na43A1
*Chaetoceros* sp. Na26B1	Na26B1
*C. tenuissimus*	GB2a
	Na26A1
	Na44A1

GB2a is a strain from the Gulf of Naples.

*Ch *Chile, *Na *Naples, *Ro *Roscoff.

### Data pre-processing for single-strain HTS

From raw fastq data, adapters and primers were removed with cutadapt^44^, allowing a maximum of three mismatches. All reads with a length < 350 bp and quality score < 20 were discarded. Because data obtained with Ion Torrent technology are known to be sensitive to a high indel error rate in homopolymeric regions^45^, we corrected indel errors using ICC v2.0.1^46^.

### Data analysis

Data were analysed by means of abundance plots, analysis of similarity and phylogenetic haplotype networks. For computational and graphical reasons, we only considered for our analyses, when available, the first most abundant 50 haplotypes for both environmental and single-strain samples. As first data exploration, we plotted abundance patterns of the dominant haplotype over the minor haplotypes of validated species inferred from the environmental samples. Plots were made in R^47^ using the packages *ggplot2*^48^, *gridExtra*^49^ and *scales*^50^. Furthermore, to ascertain if the aforementioned haplotype abundance patterns in both strains and environmental samples fitted existing distribution models, we explored the Rank Abundance Distribution (RAD) models using the radfit function in *vegan*^51^. This function fits the predictions of some of the most popular species abundance models (Broken stick Null model, Preemption model, Lognormal, Zipf and Zipf-Mandelbrot) to empirical data using maximum likelihood estimation.

As second step, we explored the pattern of similarity among the validated environmental haplotypes of *Chaetoceros* species, the reference barcodes and monoclonal single-strain data for each species using BLAST^39^. In particular, we assessed if: (i) the most abundant haplotype in each single strain matched the reference barcode of that strain obtained with Sanger sequencing and with the dominant environmental haplotype; (ii) the minor haplotypes in the strains were also found in the environmental samples. Furthermore, in order to assess the efficiency of homogenisation we calculated, for each strain, the percentage of reads clustering with the dominant haplotype. Clustering was performed at the threshold of 99% of similarity based on the findings of^52^ using *vsearch* in mothur^43^.

Finally, we inferred haplotype networks for each selected *Chaetoceros* species from temporal environmental data for a graphical visualisation of concerted evolution. If this phenomenon was occurring in our target species, we expected to see a major node (the dominant haplotype) surrounded by smaller ones whose temporal pattern (colour pattern in the nodes) was consistent. Networks were inferred using the TCS method^53^ implemented in PopART v1.7^54^. Only metabarcodes with abundance ≥ 2 were used to reduce the number of sequences to be processed for network inference. Furthermore, for *C. costatus* and *C. tenuissimus*, we further reduced the number of haplotypes analysed considering only the ones with abundance ≥ 10 and ≥ 50 respectively, in order to obtain a clearer graphical visualisation of networks. Metabarcode dates were pooled together in months, and a different colour was assigned to each of them. To test if the temporal distribution of dominant and minor haplotypes in the networks was the same, we inferred the Kolmogorov–Smirnov test using PAST v3.24^55^. For the test we selected in each species’ network, whenever possible, a few peripheral nodes with a distribution of reads over the months comparable (a colour pattern similar) to that of the dominant haplotype (nodes marked with *) as well as peripheral nodes with a markedly different distribution (nodes marked with #). We used 1000 Monte Carlo permutations for assessing the statistical significance of p values.

## Results

### General characteristics of the datasets

The number of reads and haplotypes (total number and sequences utilised for network analyses) for each species from the temporal environmental dataset after the validation procedure described in “Methods” section is provided in Supplementary Table S2. Briefly, the number of total validated reads ranged from 366 (90 haplotypes) in *C. anastomosans* to 139,185 (2585 haplotypes) in *C. tenuissimus*. The number of haplotypes utilised for network inference ranged from 15 (*C. anastomosans*) to 527 (*Chaetoceros* sp. Na11C3).

For single strain HTS, the number of reads ranged from 32,112 (*C. curvisetus* 2 Na1C1) to 516,766 (*Chaetoceros* sp. Na11C3) and, after pre-processing, from 19,185 (*C. curvisetus* 2 Na1C1) to 94,449 (*Chaetoceros* sp. Na11C3). The number of haplotypes used for following analyses ranged from a minimum of 2002 (*C. curvisetus* 2 strain Na1C1) to a maximum of 4696 (*C. costatus* strain Na32B1) (Supplementary Table S3). Raw data relative to single strain HTS can be found in NCBI Sequence Read Archive (SRA) at the accession numbers SAMN15700870–SAMN15700885.

### Abundance plots from environmental metabarcoding and single strain HTS

**Figure 1:**
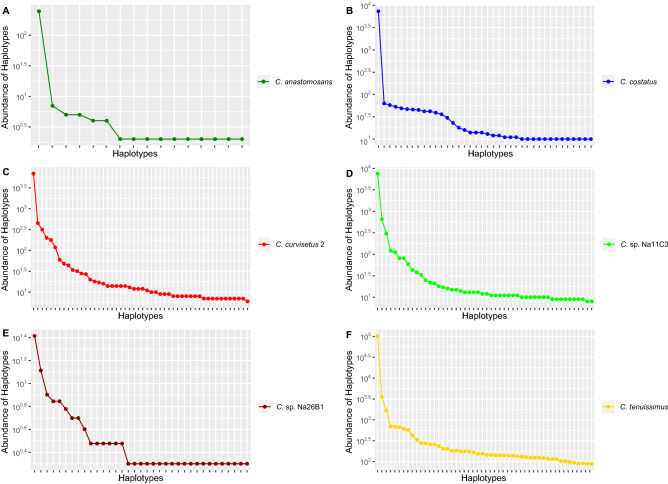
Abundance plots for each *Chaetoceros* species from validated environmental sequences. (**A**) *C. anastomosans*; (**B**) *C. costatus*; (**C**) *C. curvisetus* 2; (**D**) *Chaetoceros* sp. Na11C3; (**E**) *Chaetoceros* sp. Na26B1; (**F**) *C. tenuissimus*. Only the first 50 most abundant haplotypes, when available, were plotted in decreasing order of read number per haplotype. Data were from the temporal metabarcoding dataset “MareChiara” (January 2011 to December 2013).

**Figure 2:**
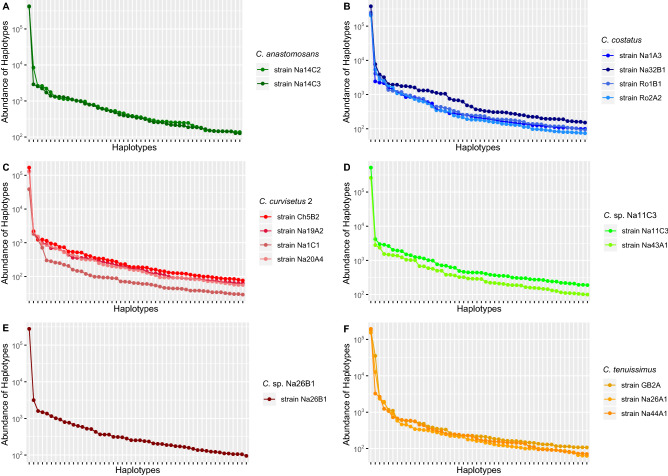
Abundance plots for each strain analysed in different *Chaetoceros* species. (**A**) *C. anastomosans*; (**B**) *C. costatus*; (**C**) *C. curvisetus* 2; (**D**) *Chaetoceros* sp. Na11C3; (**E**) *Chaetoceros* sp. Na26B1; (**F**) *C. tenuissimus*. Data are from single strain high throughput sequencing. Only the first 50 most abundant haplotypes were plotted.

The plotting of the 50 most abundant haplotypes (Supplementary Table S4) from environmental metabarcoding data versus their abundance in each species (Fig. 1) showed a characteristic pattern. Indeed, in each species analysed, of all the haplotypes attributed to a particular species (environmental samples) there was one (the “dominant haplotype”) that was far more abundant of all the others, of at least one order of magnitude (Fig. 1). All the other copies occurred in the environment at lower abundance. Patterns of abundance distribution in the HTS of single strains showed the same trend observed in the matabarcoding data of environmental samples (Fig. 2). Indeed, in each strain there was the same steep decrease in abundance from the dominant haplotype to the most abundant minor haplotype and then the more or less linear decrease along the minor haplotypes when scaled logarithmically. Furthermore, within the same species, the distribution of the 50 most abundant haplotypes among strains was congruent (Fig. 2) and most of minor haplotypes, despite not in the same ranking order, were identical across strains in this threshold (Supplementary Table S5). The comparison of models using Bayesian Information Criteria (BIC) highlighted in all the cases a preference for the Zipf model (Supplementary Figs. S1 and S2). Based on this model, the abundance of a haplotype was found to be inversely proportional to its rank and the expected abundance (a) of a haplotype at rank r is: ar = N · p1 · rγ, where N is number of reads, p1 is the fitted proportion of the most abundant haplotype, and γ is a decay coefficient. The Zipf-Mandelbrot model, a derivative of the Zipf model, was equally fitting our data (Supplementary Figs. S1 and S2).

### Analyses of similarity

For each species, the reference barcode (obtained with Sanger sequencing) matched at 100% of similarity with the dominant haplotypes of environmental data and single strain HTS (Supplementary Table S6). In most of the species here examined, more than half of validated environmental minor haplotypes were also found in single strains HTS at 100% of similarity. Overall, a match between environmental and single strain haplotypes was found for each species within the similarity threshold of 99.20% (Table 2). The clustering analysis conducted in each strain to test the efficiency of homogenisation process showed that ca. 99% of the reads clustered with the dominant haplotype ≥ 99% similarity (Supplementary Table S7).

**Table 2:** Cumulative percentage of all environmental haplotypes found in the pooled single strains of each *Chaetoceros* species divided in classes of similarity.

Species	N environmental haplotypes	% similarity with haplotypes found in strains			
		100	99.74–99.73	99.48–99.47	99.21–99.20
*C. anastomosans*	14	42.9	92.9	100	–
*C. costatus*	38	73.7	100	–	–
*C. curvisetus* 2	369	53.6	95.1	100	–
*C.* sp. Na11C3	527	56.9	95.2	99.4	100
*C.* sp. Na26B1	59	45.8	95	100	–
*C. tenuissimus*	102	60.8	100	–	–

### Phylogenetic networks from environmental samples

**Figure 3:**
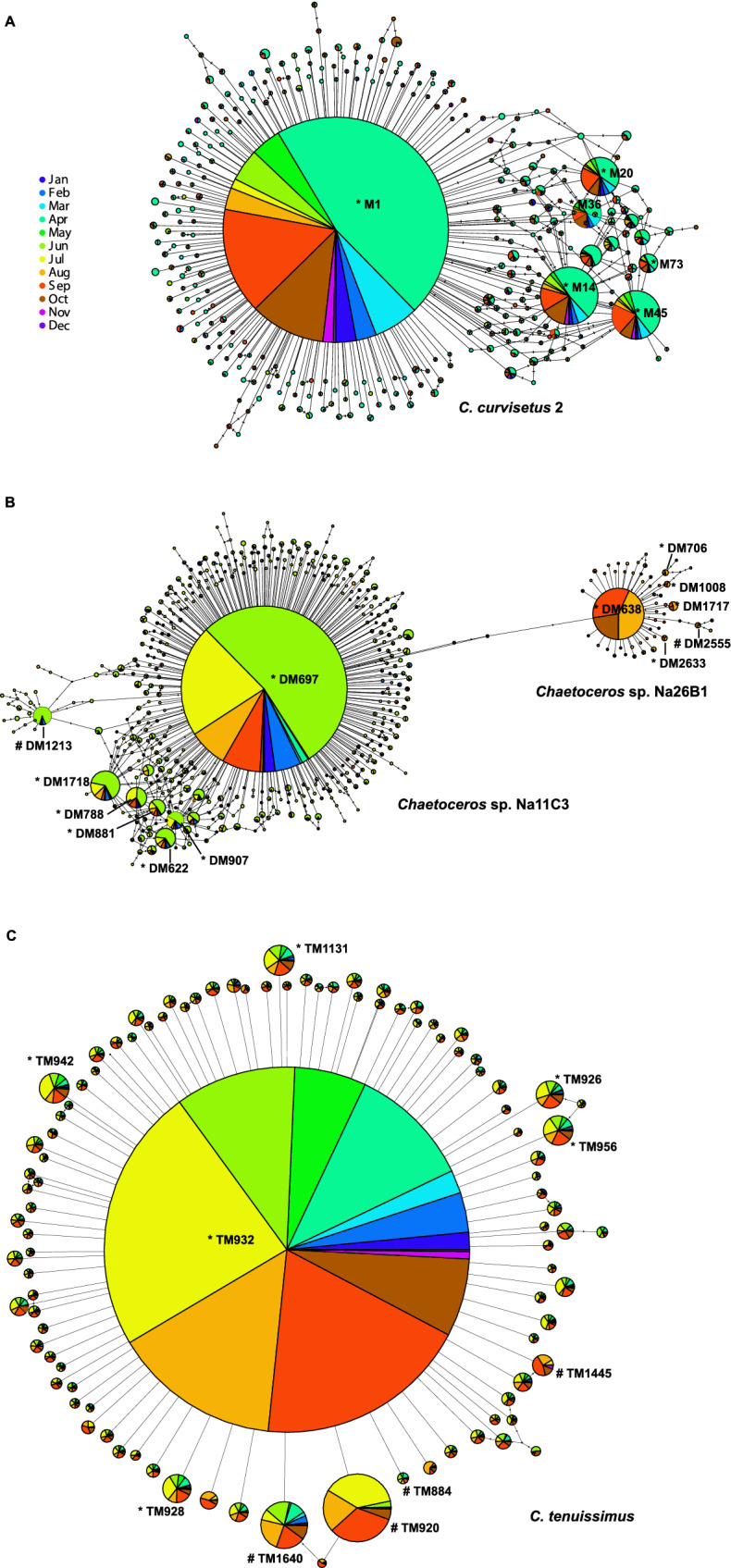
TCS haplotype networks inferred from environmental metabarcoding data. (**A**) *C. curvisetus* 2; (**B**) *Chaetoceros* sp. Na11C3 and *C.* sp. Na26B1; (**C**) *C. tenuissimus*. Haplotypes are grouped per month across 2011 and 2013 and partitioned as follows: 369 haplotypes (abundance ≥ 2) for *C. curvisetus* 2, 527 haplotypes for *C*. sp. Na11C3 and 59 for *C.* sp. Na26B1 (abundance ≥ 2) and 103 haplotypes (abundance ≥ 50) for *C. tenuissimus*.

In all the species here analysed with a sufficient number of haplotypes (Fig. 3), the temporal pattern observed in the node containing the dominant haplotype corresponded with the temporal pattern of the other nodes containing haplotypes with lower abundance. This was particularly straightforward for *C. curvisetus* 2 (Fig. 3A), *Chaetoceros* sp. Na11C3 (Fig. 3B) and *C. tenuissimus* (Fig. 3C). These were also the species with the highest number of haplotypes utilised (369, 527 and 103 respectively). In *C. anastomosans* (Supplementary Fig. S3) this pattern is almost absent due to the low number of reads validated from the MareChiara dataset. In *C. tenuissimus* (Fig. 3C) the pattern of concerted evolution is particularly evident, which relates to the fact that it was the species with the highest number of haplotypes. Indeed, almost all the nodes around the central one containing the dominant haplotype showed a temporal pattern mimicking it. The inclusion of only the first 50 most abundant haplotypes in the analysis reduced the noise due to haplotypes at low read-abundances (e.g. less than 10) as observed in the networks of species with less abundant overall read numbers. As expected, the Kolmogorov–Smirnov test confirmed that the selected minor haplotypes with a read distribution over the months similar to that of the dominant haplotype (similar colour pattern) did not deviate significantly from that of the dominant haplotype (p > 0.05, Supplementary Table S8), whereas those with a strikingly different colour pattern deviated significantly (p < 0.05, Supplementary Table S8).

## Discussion

Thanks to the experimental design presented in this study, we have demonstrated that the 18S rDNA region is under concerted evolution in the *Chaetoceros* species here analysed. Our results suggest that homogenisation is highly efficient at maintaining nearly identical 18S rDNA repeats. However, homogenisation remains incomplete as shown by the presence of minor haplotypes, which falls within the threshold of similarity of 99% in respect to the dominant haplotype. These results suggest that the 18S rDNA is evolving via concerted evolution rather than birth-and-death evolution^5^. Indeed, in studies reporting the occurrence of birth-and-death evolution, a greater genetic divergence among rDNA copies (around 10–15%) is observed^56–59^, as consequence of the fact that some copies are randomly maintained in the genome for a long time while others are deleted^5^. Furthermore, we demonstrate that minor haplotypes found in the environmental samples are no technical artefacts because these same haplotypes are encountered not only in independently analysed samples from the same collection site but also in single strains within the threshold of 99.5% of similarity. We do not exclude that part of the minor variation is due to sequence error, but sequence error cannot explain the same sets of minor haplotypes all over the 48 independent samples and in the independently analysed strains.

The graphs obtained by plotting the first fifty most abundant haplotypes in temporal and single-strain HTS data also confirm the occurrence of concerted evolution. Indeed, both haplotypes from environmental metabarcoding and single strain HTS exhibit the same distribution pattern, with one haplotype that is far more abundant than all the others by at least one order of magnitude (the “dominant haplotype”), followed by other lowly abundant haplotypes (“minor haplotypes”). The fact that within the same species different strains share most of the minor haplotypes (though not in the same ranking order) is explained by the fact that concerted evolution requires not only the horizontal transfer of mutations among the repeats (homogenisation), but also the spread of mutations to all the individuals in the population (fixation)^7^. Furthermore, the fact that geographically distant strains share several minor haplotypes (e.g. *C. costatus* strains from Atlantic France and Mediterranean Naples, and *C. curvisetus* 2 strains from Central Chile and Mediterranean Naples) indicate that these haplotype variants were already present in the ancestral population from which these regional populations derived.

The pattern of haplotype abundance distribution here found best fits the Zipf- and Zipf-Mandelbrot (ZM) models among the RAD models tested. Evidence for these models exist in many biological systems^60–63^. Yet, the fitted distribution correctly describes only the first part of the empirical curves in both single strain and environmental data. Based on the abundance of the dominant haplotype, the Zipf-ZM models shapes a more rapid decay of the abundance of minor haplotypes given their rank. Several reasons are probably co-responsible for this discrepancy. Concerted evolution processes work towards homogenisation, but never fully achieve it. These models ideally fit an infinitely large dataset, whereas numbers of reads per haplotype are limited by our sample size, with many haplotypes on the right side of the curve with one read. A haplotype cannot include a fraction of a read, whereas the model can. In addition, we cannot exclude PCR and sequencing artefacts. Additional work is needed to ascertain if the Zipf-ZM models represent the best models for concerted evolution in other species across the Tree of Life. For this purpose, time-series metabarcoding data could be used to spot the signal of concerted evolution. However, these preliminary findings should be confirmed by other empirical data.

The presence of one dominant haplotype per species suggest that rDNA repeats in *Chaetoceros* are arranged in a single locus, but data on chromosomal distribution or number of nucleoli are needed to confirm this hypothesis. Regarding the copy number of rDNA cistron, several studies have demonstrated that it is highly variable: from 60 to 220 copies in fungi^64^ and 39–19,300 in animals and 150–26,048 in plants^65^. Among protists, ciliates harbour the highest number of rDNA copies, between 3000 and 400,000^66^, diatoms possess between 1057 and 12,812^67^ and dinoflagellates between 200 and 1200^68^. In our study, assuming that each haplotype corresponds to a different rDNA unit, the number of repeats is around 3600 copies (min 2002; max 5055), within the values reported for diatoms. However, these estimations could overestimate the real number of copies due to the occurrence of PCR and sequencing errors. High variation among copies has been detected using a cloning and sequencing approach in fungi^69^, dinoflagellates^70,71^, and Foraminifera^28^, as well as with genome sequencing in the plant genus *Asclepias*^72^. However, the biological relevance of having many rDNA haplotypes is largely unknown. Part of such variation could simply be due to imperfection of the homogenisation mechanism. Another explanation, complementary to the former, is that there could be a selective advantage in possessing all these different copies. Indeed, in bacteria it has been shown that the number of copies of the small rDNA gene correlates with the rate at which phylogenetically diverse bacteria respond to resource availability, with a high copy number leading to rapid colony formation^73^. In eukaryotes, the copy number of rDNA genes is unstable^74^ and its stabilisation extends lifespan in yeast^75^. Always in yeast, it has been demonstrated recently that DNA replication stress induces a reduction in rDNA copy number in yeast^76^. The possible role of rDNA heterogeneity in protists is yet to be unveiled.

The results of analyses of similarity reveal that the dominant haplotypes of all the monoclonal strains analysed are identical within the same species, as well as to Sanger reference sequences and to the dominant metabarcode environmental haplotype of that species. Moreover, the minor haplotypes found in the environment data also occur in the monoclonal strains (intragenomic variation), and within the 99.5% of similarity in most of the cases. All these results provide empirical evidence for concerted evolution in these diatom species and, for the first time, by using single strain high-throughput sequencing and a metabarcoding time-series dataset together with a Sanger reference library.

The availability and the quality of Sanger reference barcode sequences is particularly important in the current “big biodiversity data” era, in which hundreds of millions of sequences can be generated during a single high-throughput sequencing run and the ability to check individual sequences is severely limited^77^. In the case of a multigene family subjected to effective concerted evolution, all the copies within a genome are expected to be identical and represented by the Sanger sequenced reference sequence. However, intragenomic sequence variation in multigene families is common in several eukaryotic lineages^19,69,78–80^, and sometimes involves size variation^81,82^. In these cases, a Sanger sequence can be considered as a consensus of all the divergent copies of the amplified gene. In this “consensus sequence”, most of the contribution is carried by the most abundant sequence, and therefore the Sanger sequence will read as the dominant haplotype.

Temporal metabarcoding datasets are classically used in ecological context to monitor biodiversity changes over time^38,83,84^. However, their potential goes beyond such purposes. In this study, we show that such datasets can be used also to track and test evolutionary or biological phenomena by the application of appropriate approaches. Indeed, through the inference of phylogenetic haplotype networks we obtain a graphical visualisation of concerted evolution in the *Chaetoceros* species here investigated. Each species network shows a star-shaped structure generated by intragenomic variation (lowly abundant divergent sequences corresponding to minor haplotypes) surrounding the dominant haplotype. Most of these nodes mimic the same temporal trend (seasonality) of the node containing the dominant haplotype, confirming the same origin and in accordance with the expectations of homogenisation process. Nevertheless, part of minor haplotypes shows deviations from this shared pattern. Such deviations, identified with a statistical test, can be mainly ascribed to artefacts due to PCR errors or by-product of massive parallel sequencing, even if a biological significance cannot be excluded and could be further explored.

Another novelty of our study is the use of single strain HTS data instead of clone libraries to study intragenomic variation and, specifically, concerted evolution. For example, the V4 region in the 18S gene is the currently recommended barcoding region for protists^25^, whilst the ITS region serves as such for fungi^26^. Some authors^85–87^ have argued that the concerted evolution process, known to affect ribosomal genes, may not be sufficiently effective to ensure complete sequence homogeneity. Therefore, knowing the extent of infraspecific variation and modality of evolution of such regions is vital to barcoding studies. Studies targeting the ribosomal genes in different organisms revealed the occurrence of several different copies within each organism analysed and highlighted the potential risk for barcoding studies^29,88^. Indeed, one of the characteristics of a good DNA barcode is to have high interspecific divergence and low intraspecific variability^89^. Dakal et al.^88^ argued that the presence of several haplotypes within an individual shortens the barcoding gap and should be taken into consideration in barcoding studies of yeasts. However, what is lacking in these studies is information about the abundance of these “alternative” rDNA copies. Pillet et al.^28^ tried to predict the number of haplotypes in each specimen of *Elphidium macellum* (Foraminifera) correlating the number of clones screened with the number of haplotypes found. The authors argued that although some of less abundant haplotypes could be due to PCR artefacts, the high Spearman correlation coefficient suggested that the real number of haplotypes in each individual was underestimated^28^. In this study, we demonstrate that within each strain of several *Chaetoceros* species occur thousands of 18S haplotypes, one of which is far more abundant that all the others (the “dominant” haplotype). Because of such huge differences in abundance, the probability that a “minor” haplotype is sequenced with Sanger chemistry is almost null. In turn, this means that there is no risk associated to the use of the rDNA cistron as target gene in classical DNA barcoding studies. However, in metabarcoding studies these minor haplotypes (intragenomic variation) can create a false rare diversity and therefore produce artefacts in diversity assessments^90^. This study also confirms, through the use of single strain HTS, the finding of^37^ from environmental samples, i.e. that the most abundant haplotype that is recovered for each species corresponds to the sequence that would be obtained by Sanger sequencing. Therefore, in case of a taxon for which a reference sequence is not available yet, the dominant haplotype retrieved from a metabarcoding dataset can be considered as such, and subsequently validated using Sanger sequencing when the specimen has been sampled.

In conclusion, in this study we report the occurrence of concerted evolution in several *Chaetoceros* species through a specific experimental design based on plots of haplotype distribution, analyses of sequence similarity and evolutionary networks using Sanger reference sequences, environmental time-series metabarcoding data and single strain HTS. This approach is novel with respect to the classical one, in which concerted evolution is typically inferred indirectly from phylogenetic inferences^21,32,91,92^. We also show a novel use of metabarcoding and HTS data that goes beyond the traditional ecological applications. On the one hand, we confirm that the dominant haplotype perfectly matches with the Sanger reference sequence, validating the use of the metabarcoding technique for ecological studies. On the other hand, we highlight that the high number of sequences occurring at low abundances (minor haplotypes) inflate the diversity assessments, but they are intragenomic variation occurring in the strains. In this study, we show that at 99% of similarity, all infraspecific variability is collapsed together with the dominant haplotype. This is true for *Chaetoceros*, but the validity across other genera is to be tested yet. Finally, our study is also a first attempt to fit the biological phenomenon of concerted evolution to the most popular species abundance models. A possible course of action for future research could be to compare the results obtained in this study in *Chaetoceros* with other diatom and protist species, in order to understand the evolution of such gene region as well as the applicability of metabarcoding and high throughput sequencing in ecological and evolutionary studies in other marine organisms.

## Supplementary Information

Supplementary Figure S1.

Supplementary Figure S2.

Supplementary Figure S3.

Supplementary File S1.

Supplementary Table S1.

Supplementary Table S2.

Supplementary Table S3.

Supplementary Table S4.

Supplementary Table S5.

Supplementary Table S6.

Supplementary Table S7.

Supplementary Table S8.

## Data Availability

Fastq files relative to single strain HTS are available on the Sequence Read Archive (SRA) database at the accession numbers SAMN15700870–SAMN15700885. Input data for phylogenetic haplotype networks are available on fighshare at 10.6084/m9.figshare.13299191. All other information is provided in the Supplementary Information file attached to this manuscript.
