# Supplementary material for: Empirical evidence for concerted evolution in the 18S rDNA region of the planktonic diatom genus Chaetoceros
Source: Sci Rep. 2021 Jan 12;11:807. doi: 10.1038/s41598-020-80829-6 (PMC7804092; doi:10.1038/s41598-020-80829-6)

Supplementary Information for:

Empirical evidence for concerted evolution in the 18S rDNA region of the planktonic diatom genus *Chaetoceros*

Daniele De Luca*, Wiebe H.C.F. Kooistra, Diana Sarno, Elio Biffali, Roberta Piredda*

* Authors for correspondence: Daniele De Luca (daniele.deluca088@gmail.com); Roberta Piredda (robpiredda@gmail.com)

Supplementary Figure S1. Fit distributions of environmental data to rank abundance models. Blue dots refer to empirical data.

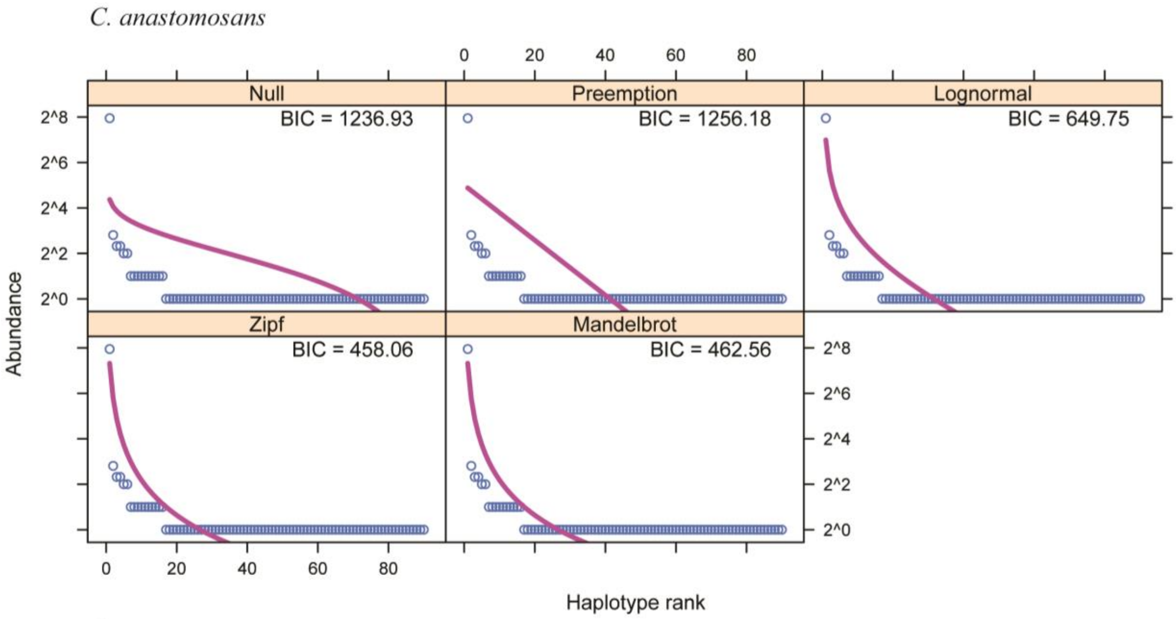

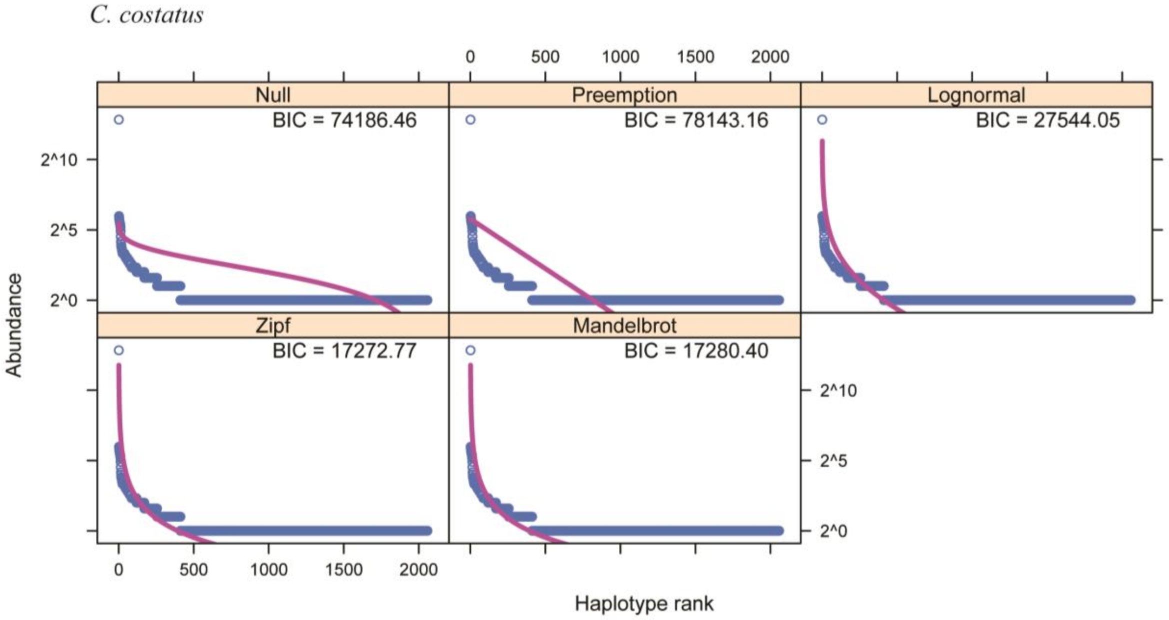

C. curvisetus 2

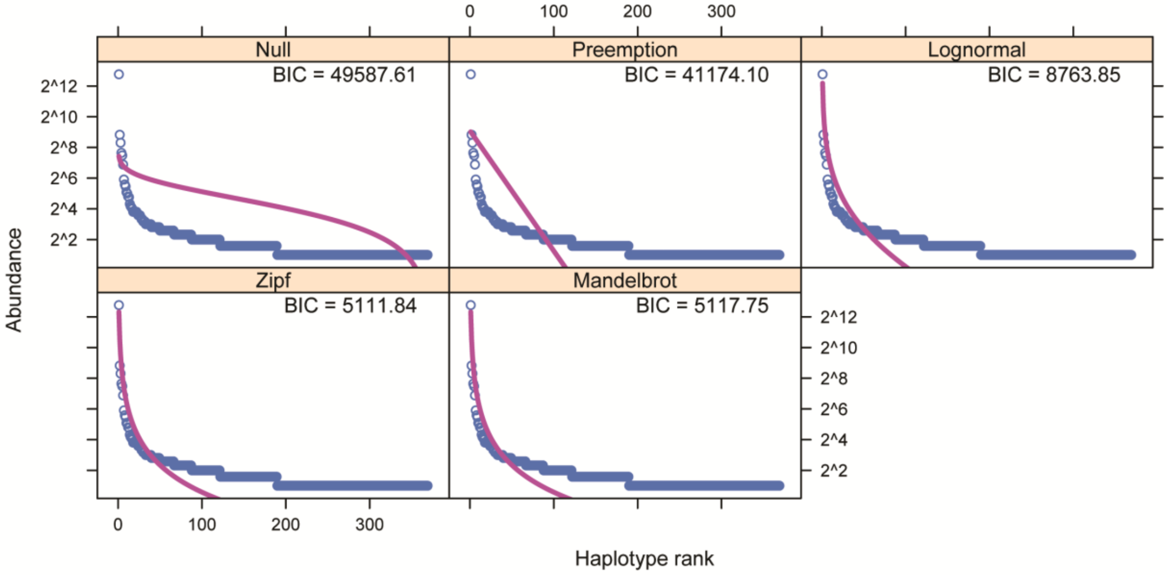

Chaetoceros sp. Na11C3 - Na26B1

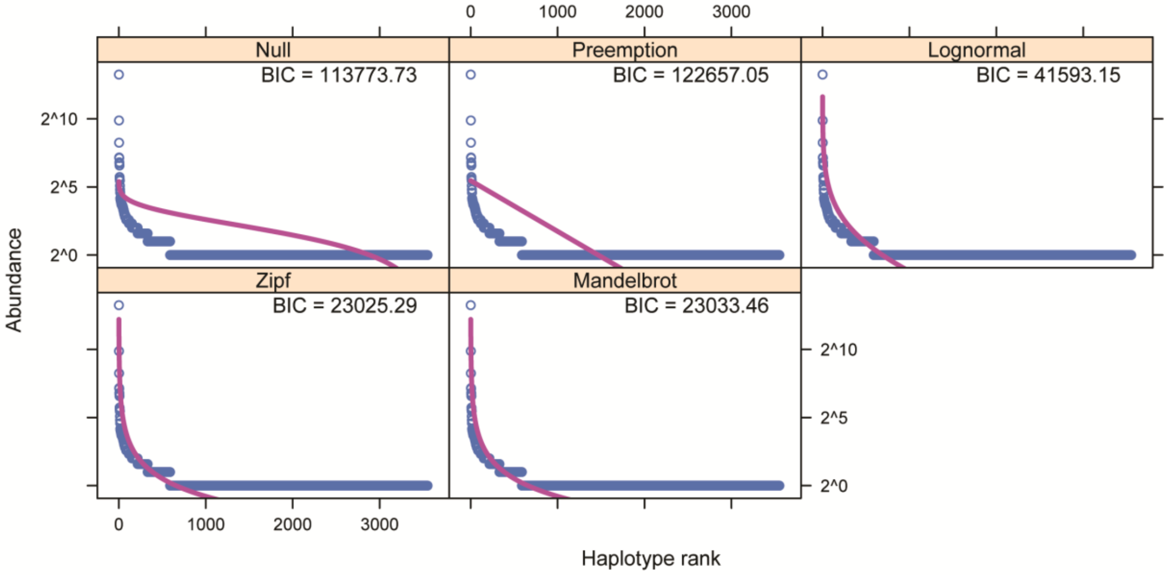

C. tenuissimus

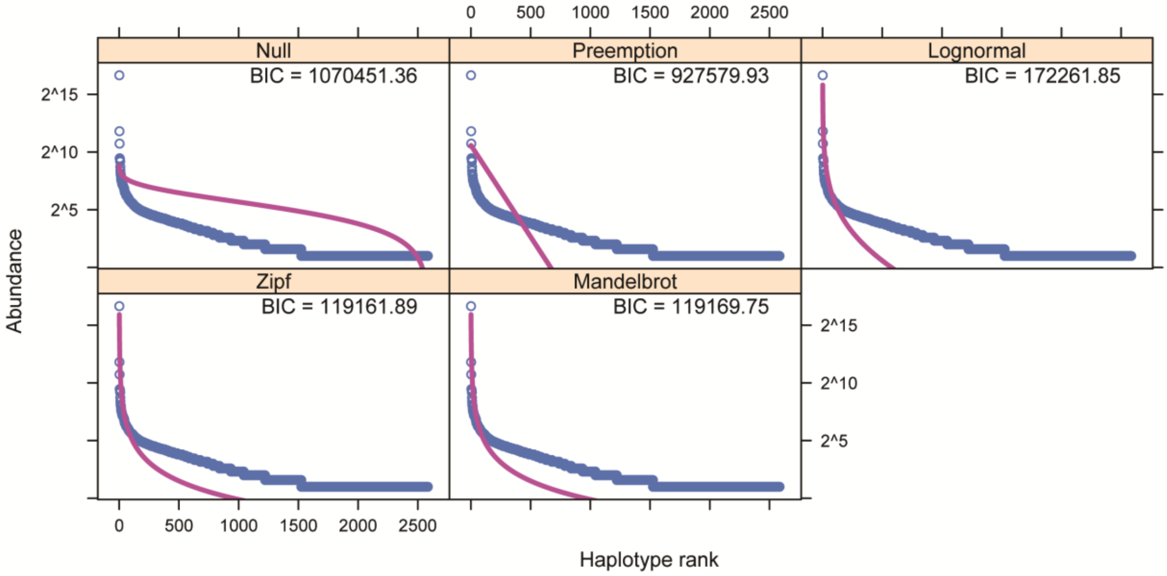

Supplement: Supplementary file 1 — Supplementary Figure S1. [file 41598_2020_80829_MOESM1_ESM.pdf]
