# Supplementary material for: Empirical evidence for concerted evolution in the 18S rDNA region of the planktonic diatom genus Chaetoceros
Source: Sci Rep. 2021 Jan 12;11:807. doi: 10.1038/s41598-020-80829-6 (PMC7804092; doi:10.1038/s41598-020-80829-6)

Supplementary Information for:

Empirical evidence for concerted evolution in the 18S rDNA region of the planktonic diatom genus *Chaetoceros*

Daniele De Luca*, Wiebe H.C.F. Kooistra, Diana Sarno, Elio Biffali, Roberta Piredda*

* Authors for correspondence: Daniele De Luca (daniele.deluca088@gmail.com); Roberta Piredda (robpiredda@gmail.com)

Supplementary Figure S2. Fit distributions of single strain data to rank abundance models. Blue dots refer to empirical data.

C. anastomosans Na14C2

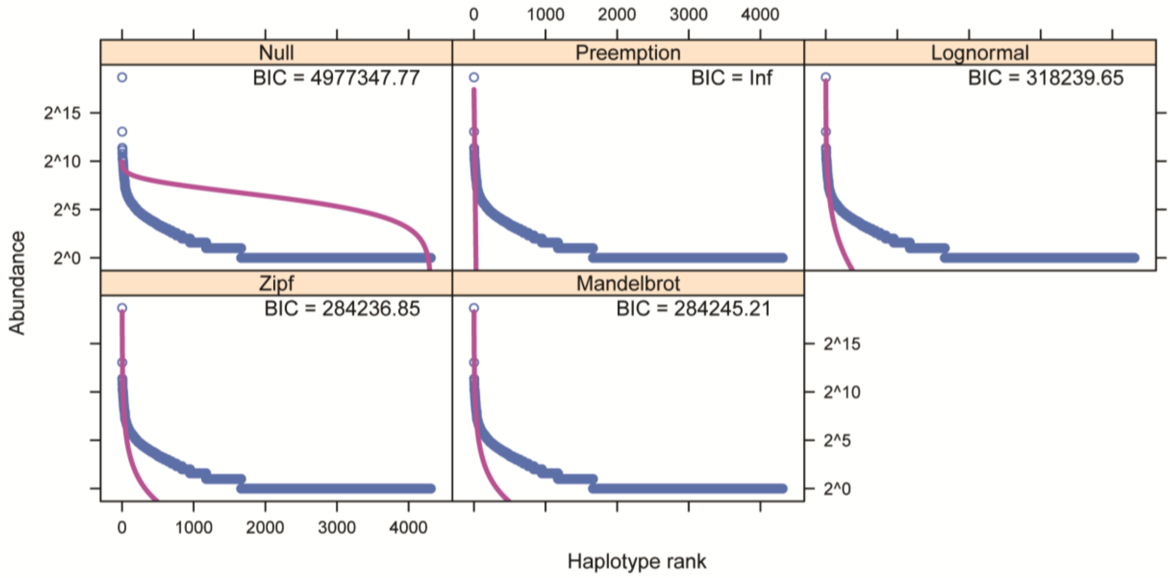

C. anastomosans Na14C3

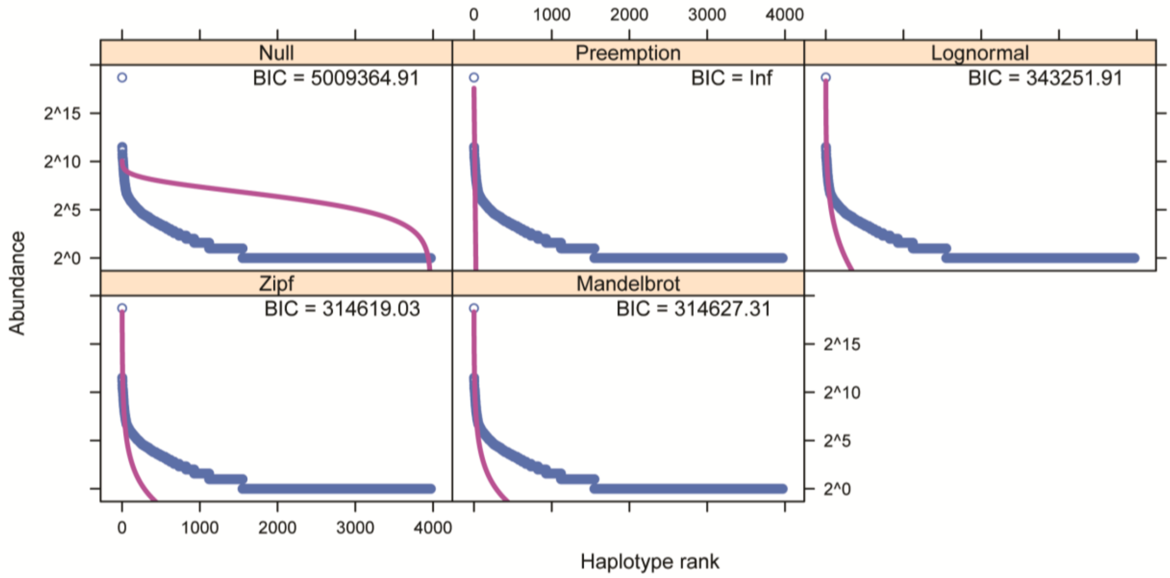

C. costatus Na1A3

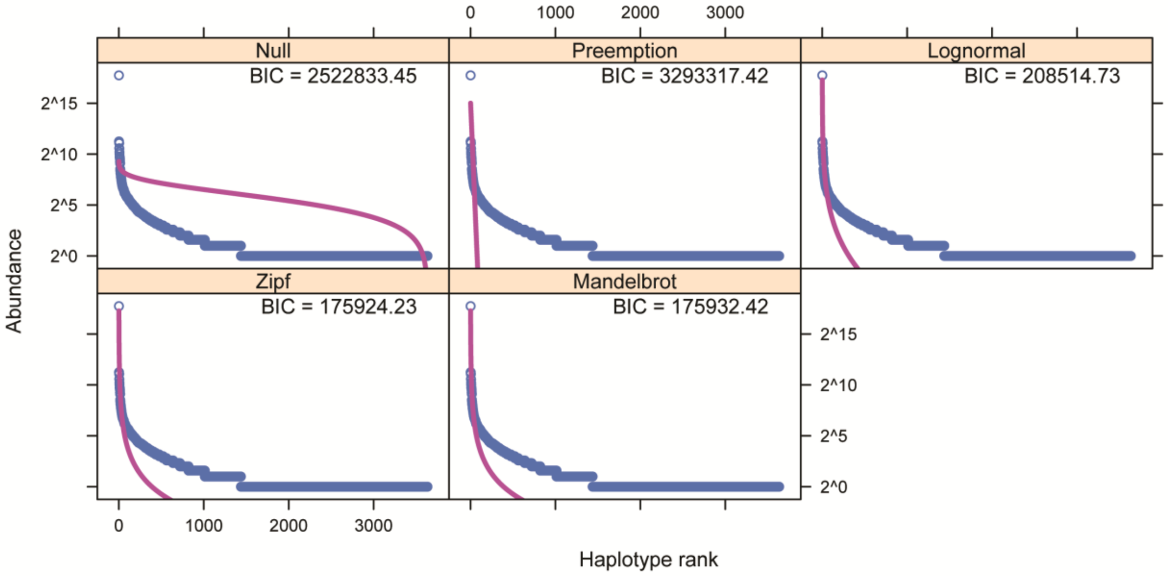

C. costatus Na32B1

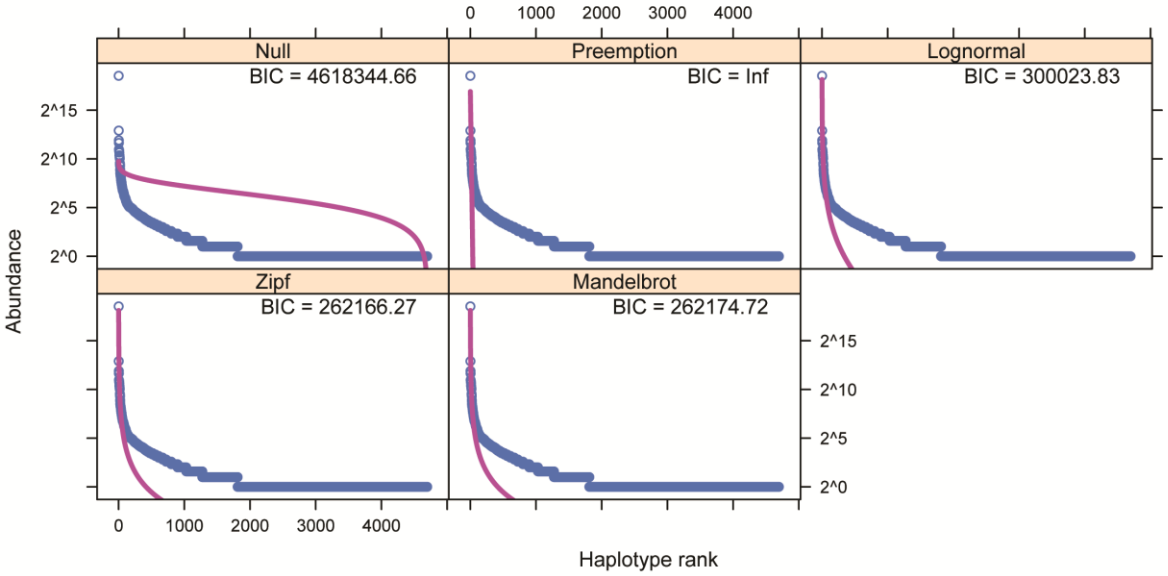

C. costatus Ro1B1

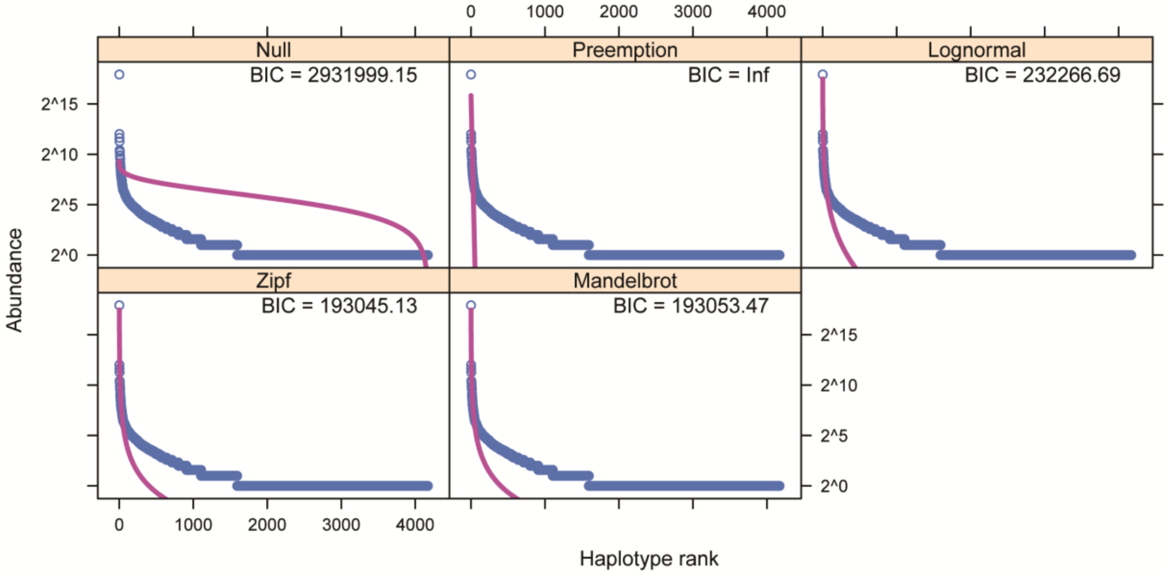

C. costatus Ro2A2

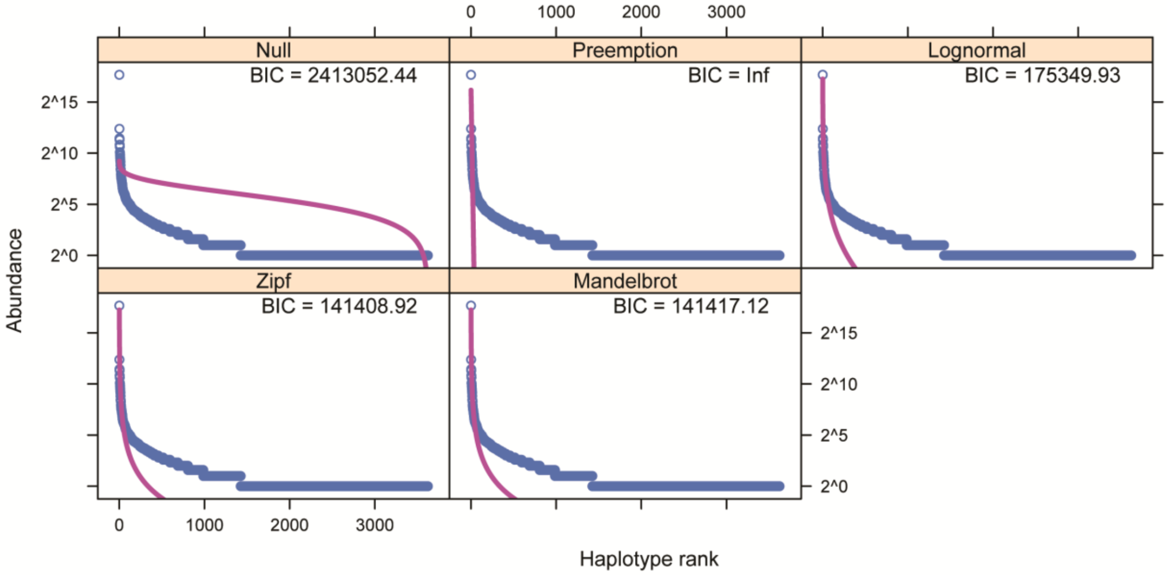

C. curvisetus 2 Ch5B2

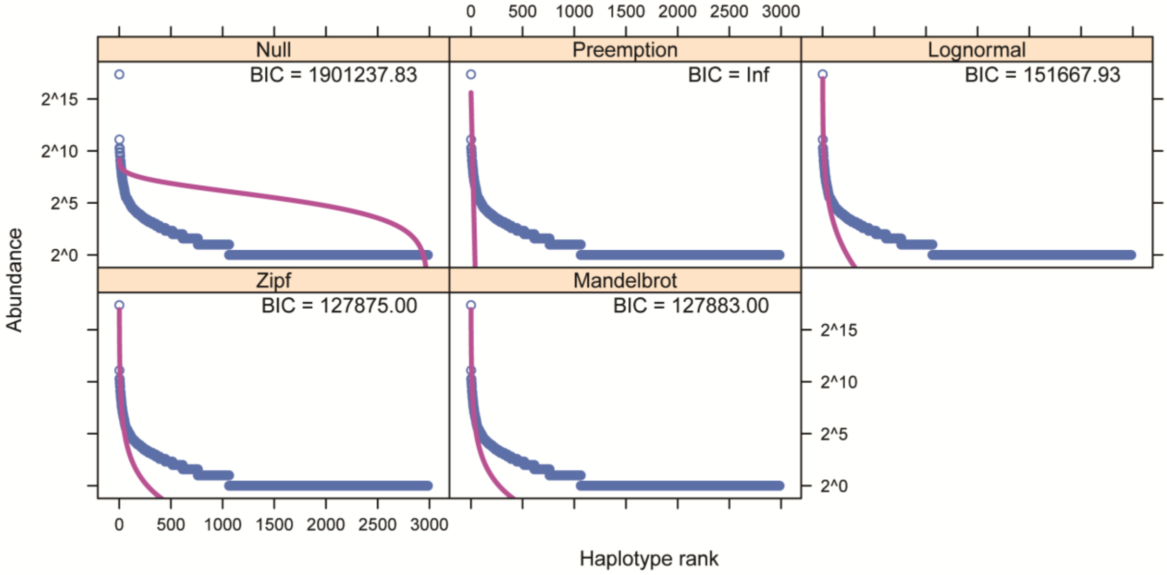

C. curvisetus 2 Na1C1

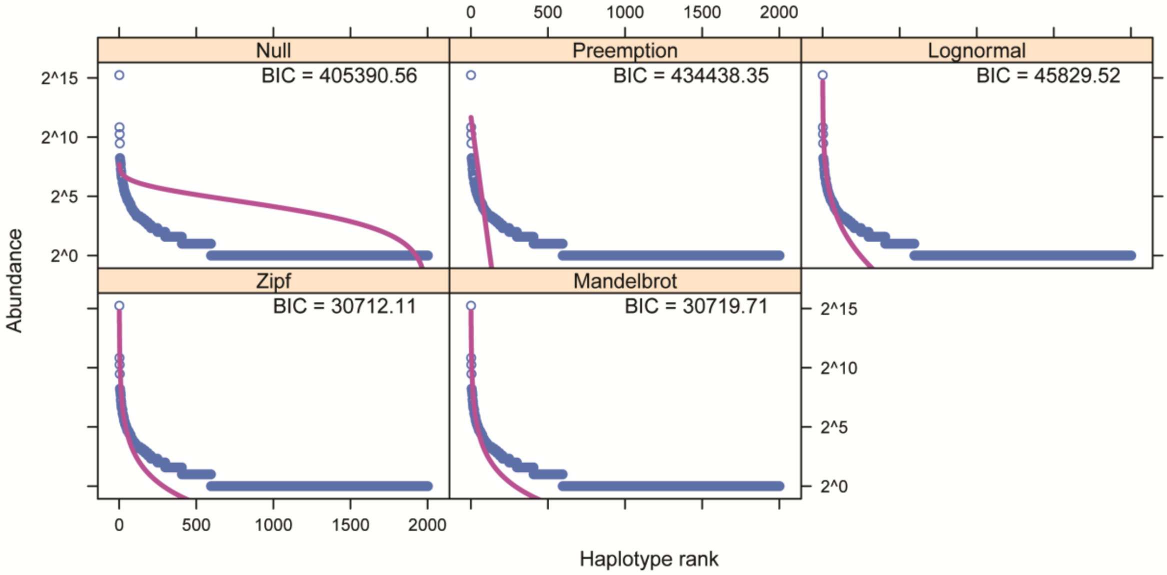

C. curvisetus 2 Na19A2

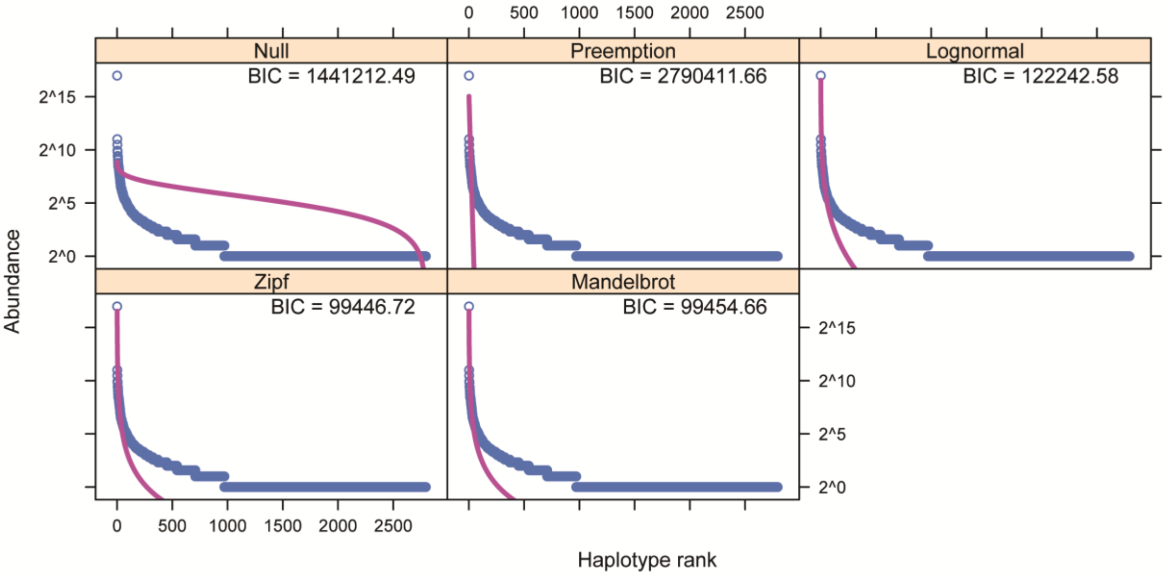

C. curvisetus 2 Na20A4

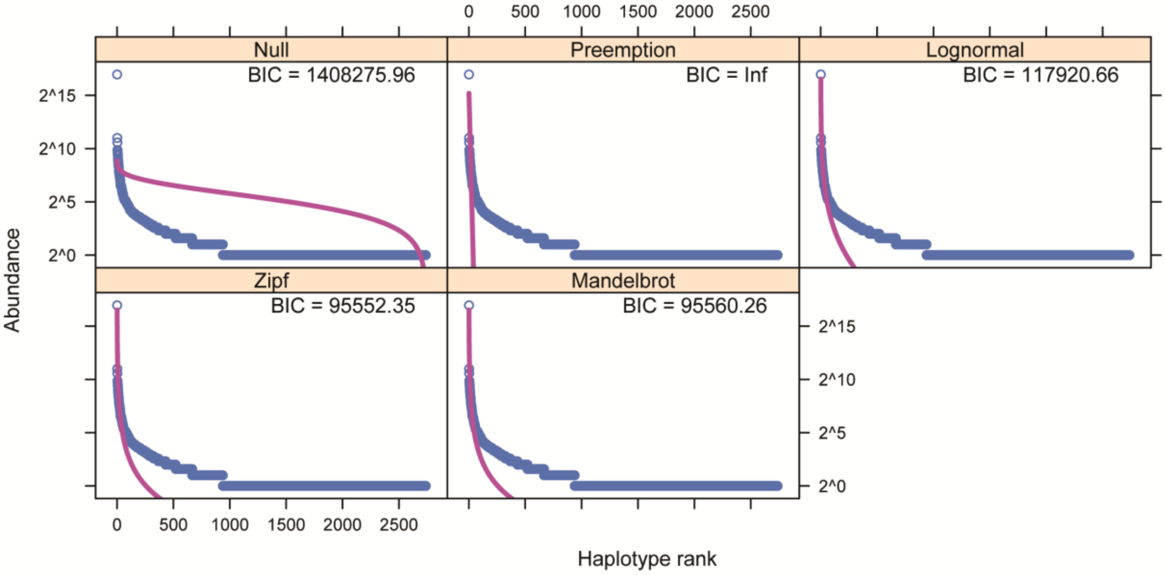

Chaetoceros sp. Na26B1

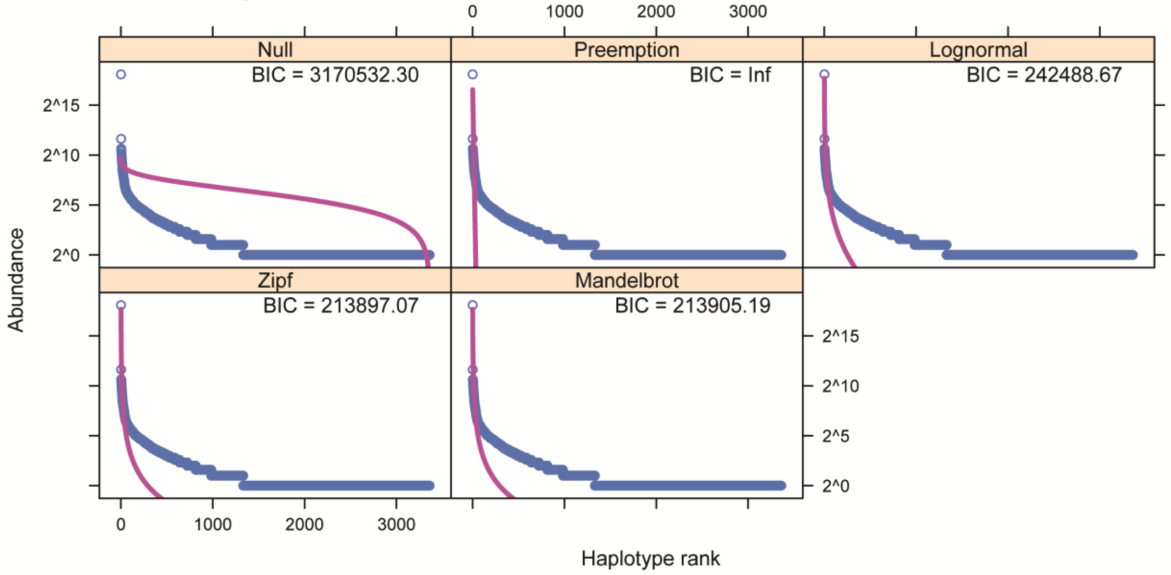

Chaetoceros sp. Na11C3 strain Na11C3

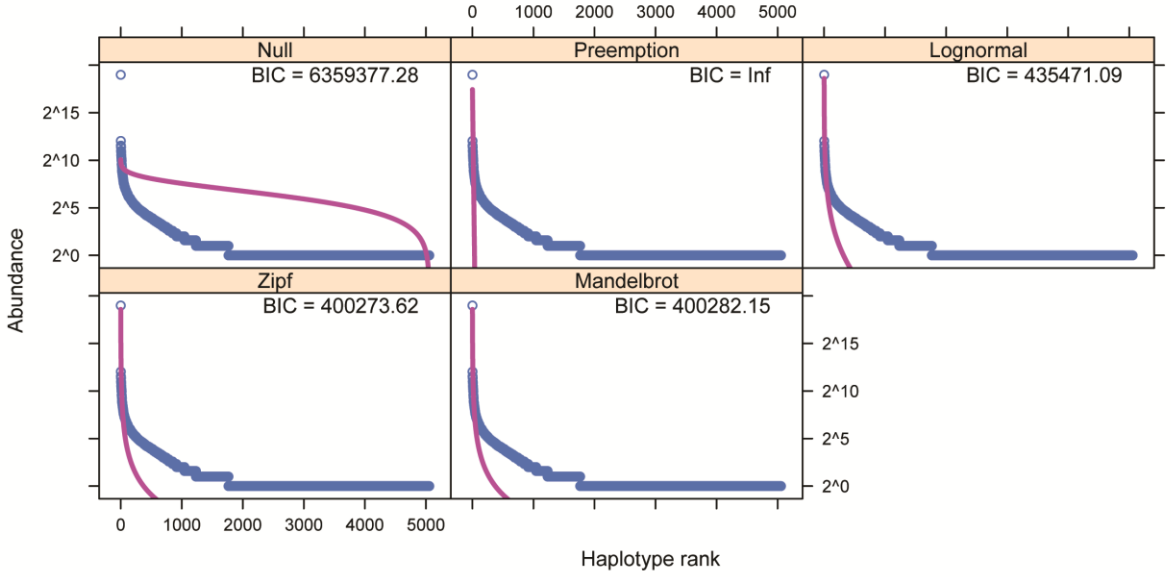

Chaetoceros sp. Na11C3 strain Na43A1

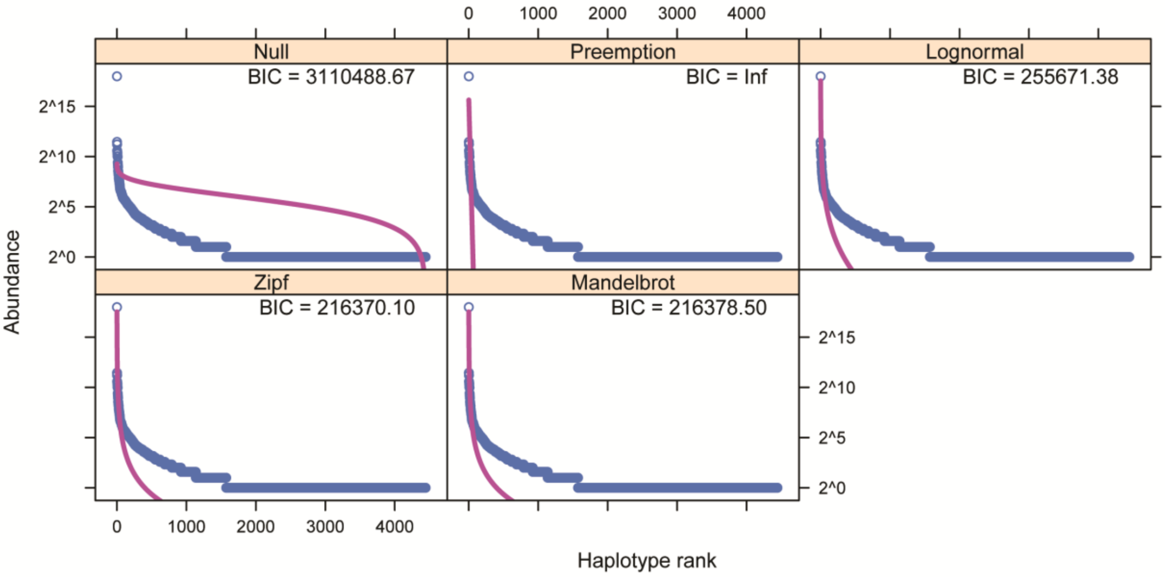

C. tenuissimus GB2a

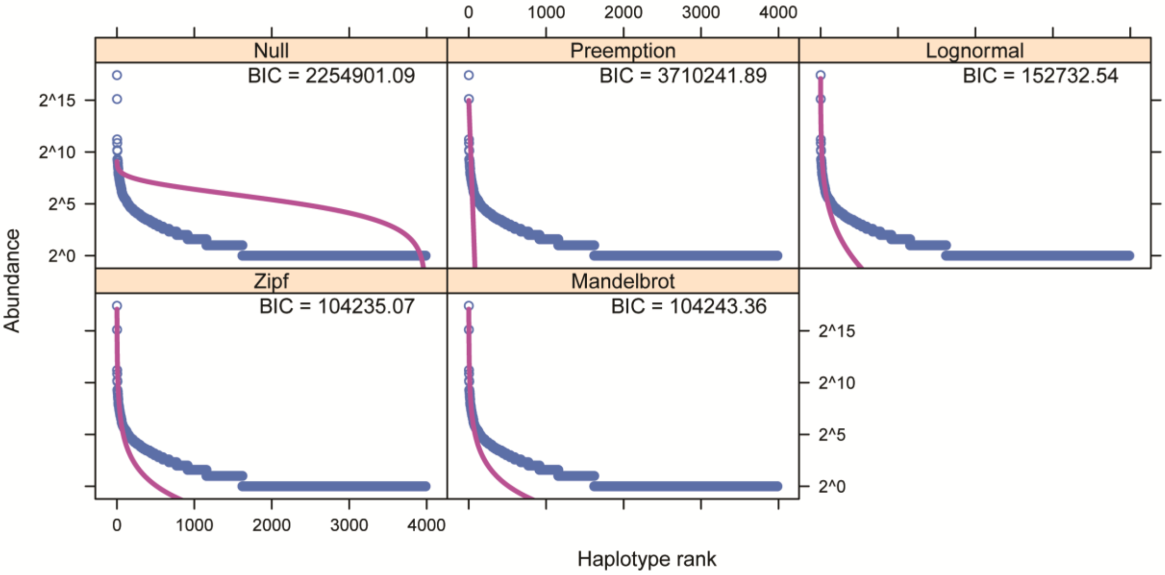

C. tenuissimus Na26A1

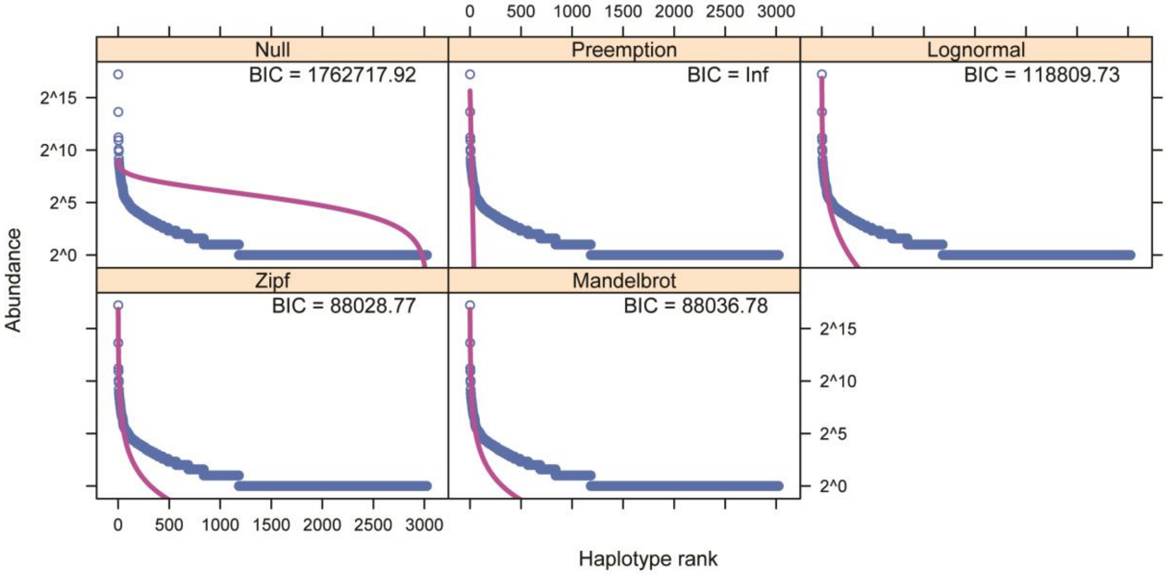

C. tenuissimus Na44A1

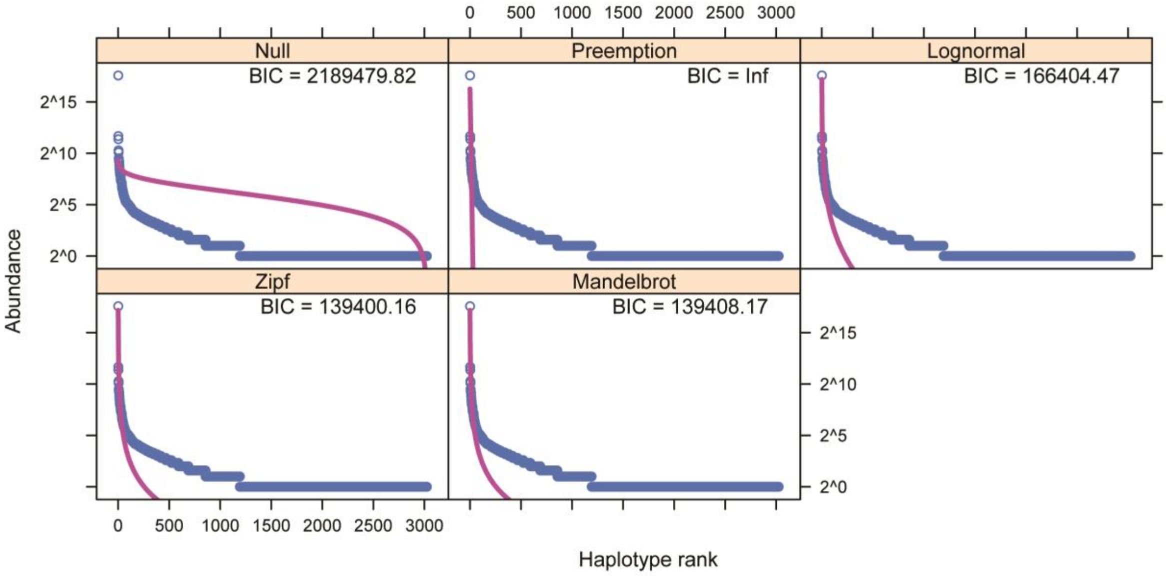

Supplement: Supplementary file 2 — Supplementary Figure S2. [file 41598_2020_80829_MOESM2_ESM.pdf]
