# Supplementary material for: Empirical evidence for concerted evolution in the 18S rDNA region of the planktonic diatom genus Chaetoceros
Source: Sci Rep. 2021 Jan 12;11:807. doi: 10.1038/s41598-020-80829-6 (PMC7804092; doi:10.1038/s41598-020-80829-6)

Supplementary Information for:

Empirical evidence for concerted evolution in the 18S rDNA region of the planktonic diatom genus *Chaetoceros*

Daniele De Luca*, Wiebe H.C.F. Kooistra, Diana Sarno, Elio Biffali, Roberta Piredda*

* Authors for correspondence: Daniele De Luca (daniele.deluca088@gmail.com); Roberta Piredda (robpiredda@gmail.com)

Supplementary Figure S3. TCS haplotype networks inferred from environmental metabarcoding data. (A) *C. anastomosans*; (B) *C. costatus*. Haplotypes are grouped per month across 2011 and 2013 and partitioned as follows: 15 haplotypes (abundance ≥ 2) for *C. anastomans* and 38 haplotypes (abundance ≥ 10) for *C. costatus*.

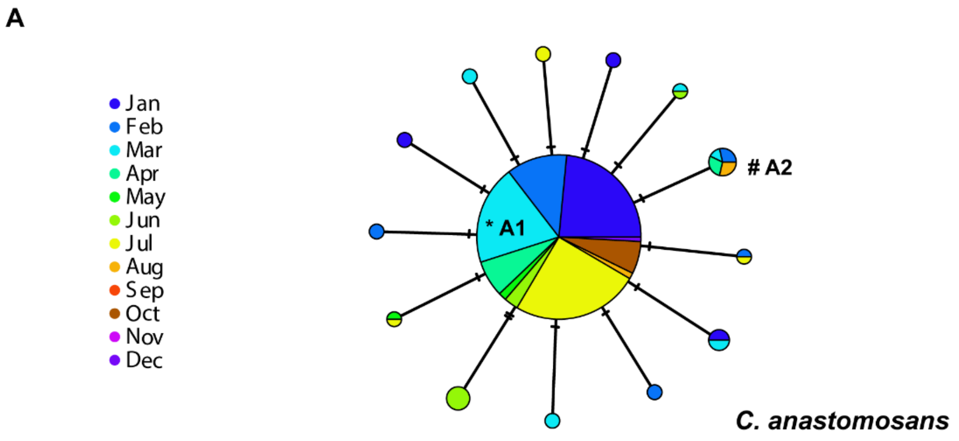

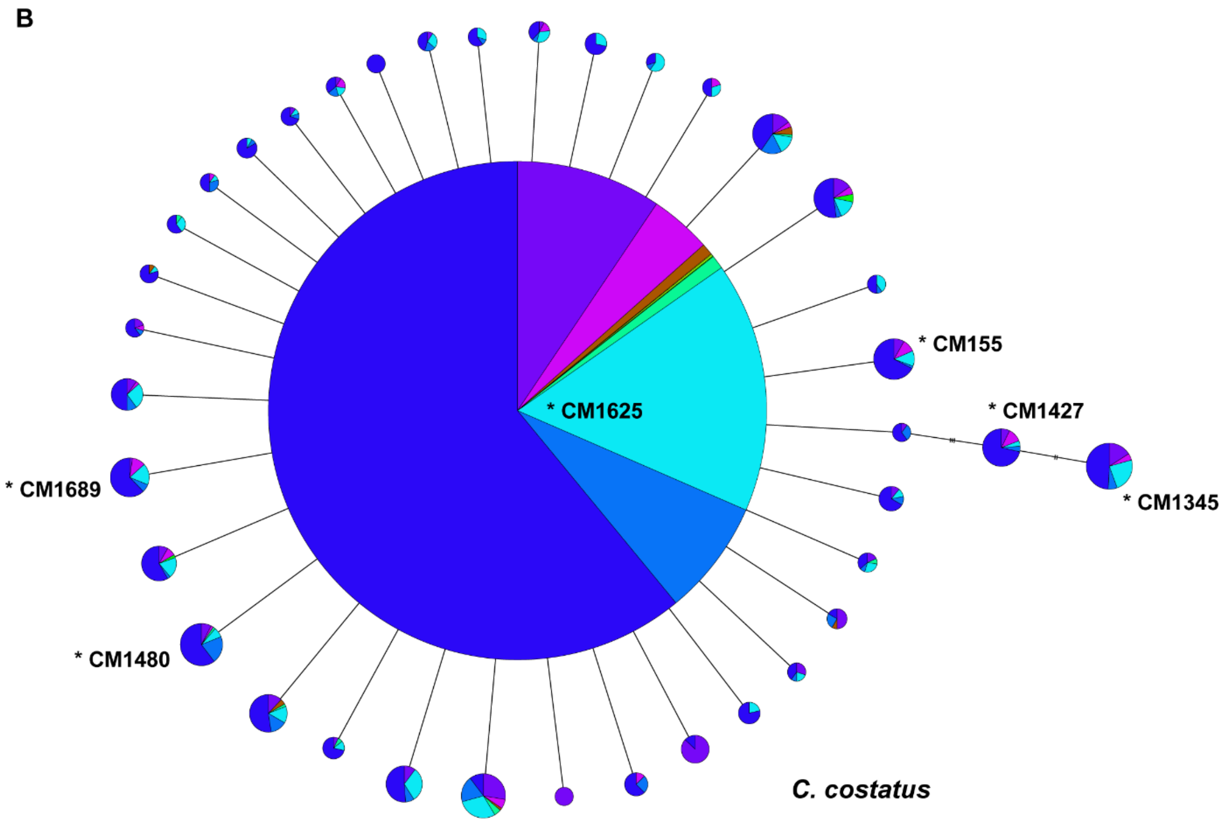

Supplement: Supplementary file 3 — Supplementary Figure S3. [file 41598_2020_80829_MOESM3_ESM.pdf]
