# Supplementary material for: Empirical evidence for concerted evolution in the 18S rDNA region of the planktonic diatom genus Chaetoceros
Source: Sci Rep. 2021 Jan 12;11:807. doi: 10.1038/s41598-020-80829-6 (PMC7804092; doi:10.1038/s41598-020-80829-6)
Supplement: Supplementary file 4 — Supplementary File S1. [file 41598_2020_80829_MOESM4_ESM.docx]

Supplementary Information for:

**Empirical evidence for concerted evolution in the 18S rDNA region of the planktonic diatom genus *Chaetoceros***

Daniele De Luca*, Wiebe H.C.F. Kooistra, Diana Sarno, Elio Biffali, Roberta Piredda*

* Authors for correspondence: Daniele De Luca (daniele.deluca088@gmail.com); Roberta Piredda (robpiredda@gmail.com)

**Supplementary File S1. Extended Materials and Methods.** PCR conditions for single strain HTS.

The first amplification was conducted using the primers targeting the 18S-V4 region by Stoeck et al. (2010) modified by Piredda et al. (2016). PCRs were conducted in a final volume of 25 µL each containing: 3 ng of DNA, 1x Buffer HF, 0.2 mM dNTPs, 0.5 μM of each primer, 1U of Phusion High-Fidelity DNA polymerase (New England Biolabs Inc, Ipswich, Massachusetts, USA) and water to volume. The thermal cycling profiles started with 98 °C for 30 s, followed by 10 cycles of denaturation at 98 °C for 10 s, annealing at 44 °C for 30 s, extension at 72 °C for 15 s, and then additional 15 cycles of denaturation at 98 °C for 10 s, annealing at 62 °C for 30 s and extension at 72 °C for 15 s, with a final extension at 72 °C for 7 min. PCR products (∼470 bp) were visualised on 1.2% agarose gel and purified using the AMPure XP Beads kit (Agencourt Bioscience Corp., Beverly, Massachusetts, USA), at a concentration of 1.2× vol/vol, according to manufacturer's instructions. The second PCR was conducted in the same volume and using the same concentrations of reagents (DNA, dNTPs, Buffer and Taq). Adapter P1 was added at a concentration of 50 µM, whilst each barcode of 20 µM. The amplification profile was as follows: initial denaturation at 98 °C for 30 s; 5 cycles of denaturation at 98 °C for 10 s, annealing at 60 °C for 30 s, extension at 72 °C for 15 s, and then a final extension at 72 °C for 7 min. The success of insertion of adapter and barcode in PCR products was checked by electrophoresis on 1.2% agarose gel (increase of size). Amplified products were purified as above and quantity and quality were determined with the Agilent DNA High Sensitivity Kit on the 2100 Bioanalyzer (Agilent Technologies, Santa Clara, California, USA) following the manufacturer’s recommendations. Since not all PCRs amplified only the fragment of interest, prior to emulsion PCR an equal amount of all COI products was pooled and processed for fragment size selection (around 500 bp). This was done by running the pooled samples on 1.2% agarose gel together with a size standard and cutting the band of interest, which was then purified using the GenElute™ Gel Extraction Kit (Sigma-Aldrich, St. Louise, Missouri, USA). Emulsion PCR was conducted in the Ion Chef System (Life Technologies, Carlsbad, California, USA) using 0.1 fmol/µL of the pool into a reaction volume of 50 µL. Massive-parallel sequencing was carried out using the Ion GeneStudio™ S5 System (Life Technologies, Carlsbad, California, USA).
