# Supplementary material for: Empirical evidence for concerted evolution in the 18S rDNA region of the planktonic diatom genus Chaetoceros
Source: Sci Rep. 2021 Jan 12;11:807. doi: 10.1038/s41598-020-80829-6 (PMC7804092; doi:10.1038/s41598-020-80829-6)
Supplement: Supplementary file 5 — Supplementary Table S1. [file 41598_2020_80829_MOESM5_ESM.docx]

Supplementary Information for:

**Empirical evidence for concerted evolution in the 18S rDNA region of the planktonic diatom genus *Chaetoceros***

Daniele De Luca*, Wiebe H.C.F. Kooistra, Diana Sarno, Elio Biffali, Roberta Piredda*

* Authors for correspondence: Daniele De Luca (daniele.deluca088@gmail.com); Roberta Piredda (robpiredda@gmail.com)

**Supplementary Table S1. List of outgroup taxa for the validation of *Chaetoceros*-species sequences.**

| **Species** | **Outgroups** | **Accession number** |
| --- | --- | --- |
| *C. anastomosans* | *C*. cf. *vixvisibilis* Na16A3  *Chaetoceros* sp. Na11C3 | MG972367  MG972328 |
| *C. costatus* | *C. cinctus* Ch6A2  *C. radicans* Ch2A2 | KY852264  KY852259 |
| *C. curvisetus* 2 | *C*. cf. *tortissimus* Na18C4  *C. tortissimus* Na25A2 | MG972275  MG972325 |
| *Chaetoceros* sp. Na11C3 / Na26B1 | *C. anastomosans* Na14C2  *C*. cf. *vixvisibilis* Na16A3  *Chaetoceros* clone HM347543 | MG972358  MG972367  HM347543 |
| *C. tenuissimus* | *C. neogracilis* 1 RCC2507  *C. neogracilis* 2 RCC2318  *C. neogracilis* 4 RCC2016  *Chaetoceros* sp.  *Chaetoceros* sp. | KT860998  JN934684  JF794049  AF145226  X85390 |
