# Supplementary material for: Empirical evidence for concerted evolution in the 18S rDNA region of the planktonic diatom genus Chaetoceros
Source: Sci Rep. 2021 Jan 12;11:807. doi: 10.1038/s41598-020-80829-6 (PMC7804092; doi:10.1038/s41598-020-80829-6)
Supplement: Supplementary file 6 — Supplementary Table S2. [file 41598_2020_80829_MOESM6_ESM.docx]

Supplementary Information for:

**Empirical evidence for concerted evolution in the 18S rDNA region of the planktonic diatom genus *Chaetoceros***

Daniele De Luca*, Wiebe H.C.F. Kooistra, Diana Sarno, Elio Biffali, Roberta Piredda*

* Authors for correspondence: Daniele De Luca (daniele.deluca088@gmail.com); Roberta Piredda (robpiredda@gmail.com)

**Supplementary Table S2. Number of environmental reads and haplotypes utilised in this study.**

| **Species** | **N reads validated**  **(N haplotypes)** | **N reads utilised for networks (N haplotypes)** |
| --- | --- | --- |
| *C. anastomosans* | 366 (90) | 287 (15) |
| *C. costatus* | 11,149 (2,057) | 8,220 (38) |
| *C. curvisetus* 2 | 11,110 (1,716) | 9,763 (369) |

| *Chaetoceros* sp. Na11C3 | 17,047 (3551)* | 12,924 (527) |
| --- | --- | --- |
| *Chaetoceros* sp. Na26B1 |  | 1,154 (59) |
| *C. tenuissimus* | 139,185 (2,585) | 121,321 (103) |

* for *Chaetoceros* sp. Na11C3 and Na26B1 reads were validated together.
