# Supplementary material for: Empirical evidence for concerted evolution in the 18S rDNA region of the planktonic diatom genus Chaetoceros
Source: Sci Rep. 2021 Jan 12;11:807. doi: 10.1038/s41598-020-80829-6 (PMC7804092; doi:10.1038/s41598-020-80829-6)
Supplement: Supplementary file 7 — Supplementary Table S3. [file 41598_2020_80829_MOESM7_ESM.docx]

Supplementary Information for:

**Empirical evidence for concerted evolution in the 18S rDNA region of the planktonic diatom genus *Chaetoceros***

Daniele De Luca*, Wiebe H.C.F. Kooistra, Diana Sarno, Elio Biffali, Roberta Piredda*

* Authors for correspondence: Daniele De Luca (daniele.deluca088@gmail.com); Roberta Piredda (robpiredda@gmail.com)

**Supplementary Table S3. Number of sequences before and after pre-processing and total number of haplotypes utilised in each strain.** Pre-processing refers to removal of adapters, primers and correction with ICC.

| **Species/strains** | **N raw sequences** | **N sequences after pre-processing** | **N haplotypes**  **after pre-processing** |
| --- | --- | --- | --- |
| ***C. anastomosans*** | | | |
| Na14C2 | 427,364 | 62,284 | 4,310 |
| Na14C3 | 431,665 | 62,183 | 3,970 |
| ***C. costatus*** | | | |
| Na1A3 | 238,922 | 34,226 | 3,634 |
| Na32B1 | 421,807 | 50,407 | 4,696 |
| Ro1B1 | 274,436 | 37,489 | 4,170 |
| Ro2A2 | 230,989 | 32,394 | 3,622 |
| ***C. curvisetus* 2** | | | |
| Ch5B2 | 161,145 | 39,735 | 2,985 |
| Na1C1 | 32,112 | 19,185 | 2,002 |
| Na19A2 | 120,545 | 34,287 | 2,794 |
| Na20A4 | 117,234 | 34,149 | 2,738 |
| ***Chaetoceros* sp. Na11C3** | | | |
| Na11C3 | 516,766 | 94,449 | 5,055 |
| Na43A1 | 259,525 | 54,973 | 4,444 |
| ***Chaetoceros* sp. Na26B1** | | | |
| Na26B1 | 273,039 | 56,985 | 3,360 |
| ***C. tenuissimus*** | | | |
| GB2a | 211,777 | 39,516 | 3,986 |
| Na26A1 | 147,806 | 34,726 | 3,024 |
| Na44A1 | 202,198 | 32,467 | 3,024 |
