# Supplementary material for: Empirical evidence for concerted evolution in the 18S rDNA region of the planktonic diatom genus Chaetoceros
Source: Sci Rep. 2021 Jan 12;11:807. doi: 10.1038/s41598-020-80829-6 (PMC7804092; doi:10.1038/s41598-020-80829-6)
Supplement: Supplementary file 10 — Supplementary Table S6. [file 41598_2020_80829_MOESM10_ESM.docx]

Supplementary Information for:

**Empirical evidence for concerted evolution in the 18S rDNA region of the planktonic diatom genus *Chaetoceros***

Daniele De Luca*, Wiebe H.C.F. Kooistra, Diana Sarno, Elio Biffali, Roberta Piredda*

* Authors for correspondence: Daniele De Luca (daniele.deluca088@gmail.com); Roberta Piredda (robpiredda@gmail.com)

**Supplementary Table S6. Correspondence between the reference barcode (Sanger sequence) of each species and the dominant haplotypes of the environmental dataset (MareChiara) and single strain HTS.** Since the reference sequences of the strains are identical to each other within the same species, only one has been chosen.

| **Species** | **Reference sequence**  **Accession number** | **Matching haplotype in MareChiara** | **% identity** | **Matching haplotype in single strain** | **% identity** |
| --- | --- | --- | --- | --- | --- |
| *C. anastomosans* | MG972358 | M00390_81_000000000-AA7DR_1_2109_10899_14476 | 100 | 97KSI_03703_04635 | 100 |
| *C. costatus* | KY852258 | M00390_81_000000000-AA7DR_1_1112_20701_25092 | 100 | 97KSI_03062_04287 | 100 |
| *C. curvisetus* 2 | MG972239 | M00390_81_000000000-AA7DR_1_1101_24335_7294 | 100 | 97KSI_04187_04119 | 100 |
| *Chaetoceros* sp. Na11C3 | MG972328 | M00390_81_000000000-AA7DR_1_1101_6410_5509 | 100 | 97KSI_03663_01512 | 100 |
| *Chaetoceros* sp. Na26B1 | MG972329 | M00390_81_000000000-AA7DR_1_1101_16198_12414 | 100 | 97KSI_01986_05212 | 100 |
| *C. tenuissimus* | MG972311 | M00390_81_000000000-AA7DR_1_1101_19390_3055 | 100 | 97KSI_00416_02071 | 100 |
