# Supplementary material for: Empirical evidence for concerted evolution in the 18S rDNA region of the planktonic diatom genus Chaetoceros
Source: Sci Rep. 2021 Jan 12;11:807. doi: 10.1038/s41598-020-80829-6 (PMC7804092; doi:10.1038/s41598-020-80829-6)
Supplement: Supplementary file 11 — Supplementary Table S7. [file 41598_2020_80829_MOESM11_ESM.docx]

Supplementary Information for:

**Empirical evidence for concerted evolution in the 18S rDNA region of the planktonic diatom genus *Chaetoceros***

Daniele De Luca*, Wiebe H.C.F. Kooistra, Diana Sarno, Elio Biffali, Roberta Piredda*

* Authors for correspondence: Daniele De Luca (daniele.deluca088@gmail.com); Roberta Piredda (robpiredda@gmail.com)

**Supplementary Table S7. Percentage of reads in each single strain HTS collapsed with the dominant haplotype at 99% of similarity.**

| **Strain** | **% reads collapsed with dominant haplotype** |
| --- | --- |
| *C. anastomosans* Na14C2 | 98.93 |
| *C. anastomosans* Na14C3 | 98.92 |
| *C. costatus* Na1A3 | 98.72 |
| *C. costatus* Na32B1 | 99.20 |
| *C. costatus* Ro1B1 | 98.87 |
| *C. costatus* Ro2A2 | 98.85 |
| *C. curvisetus* 2 Ch5B2 | 98.98 |
| *C. curvisetus* 2 Na1C1 | 99.31 |
| *C. curvisetus* 2 Na19A2 | 99.16 |
| *C. curvisetus* 2 Na20A4 | 99.26 |
| *Chaetoceros* sp. Na11C3 strain Na11C3 | 99.23 |
| *Chaetoceros* sp. Na11C3 strain Na43A1 | 98.79 |
| *Chaetoceros* sp. Na26B1 | 98.58 |
| *C. tenuissimus* GB2a | 98.81 |
| *C. tenuissimus* Na26A1 | 98.92 |
| *C. tenuissimus* Na44A1 | 98.89 |
