# Supplementary material for: Empirical evidence for concerted evolution in the 18S rDNA region of the planktonic diatom genus Chaetoceros
Source: Sci Rep. 2021 Jan 12;11:807. doi: 10.1038/s41598-020-80829-6 (PMC7804092; doi:10.1038/s41598-020-80829-6)
Supplement: Supplementary file 12 — Supplementary Table S8. [file 41598_2020_80829_MOESM12_ESM.docx]

Supplementary Information for:

**Empirical evidence for concerted evolution in the 18S rDNA region of the planktonic diatom genus *Chaetoceros***

Daniele De Luca*, Wiebe H.C.F. Kooistra, Diana Sarno, Elio Biffali, Roberta Piredda*

* Authors for correspondence: Daniele De Luca (daniele.deluca088@gmail.com); Roberta Piredda (robpiredda@gmail.com)

**Supplementary Table S8. Kolmogorov-Smirnoff test for equal distributions between dominant and selected minor haplotypes from environmental metabarcoding in *Chaetoceros* spp.** In parentheses are p values after 1000 permutations.

(a) *C. anastomosans*

| **Dominant haplotype** | **Minor haplotype** | **D value** | **p value** |
| --- | --- | --- | --- |
| A1 | #A2 | 0.6 | 0.031 (0.052) |

(b) *C. costatus*

| **Dominant haplotype** | **Minor haplotype** | **D value** | **p value** |
| --- | --- | --- | --- |
| CM1625 | *CM1345 | 0.5 | 0.111 (0.158) |
|  | *CM155 | 0.4 | 0.313 (0.336) |
|  | *CM1427 | 0.5 | 0.111 (0.14) |
|  | *CM1689 | 0.5 | 0.111 (0.144) |
|  | *CM1480 | 0.5 | 0.111 (0.156) |

(c) *C. curvisetus* 2

| **Dominant haplotype** | **Minor haplotype** | **D value** | **p value** |
| --- | --- | --- | --- |
| M1 | *M14 | 0.25 | 0.786 (0.853) |
|  | *M45 | 0.167 | 0.991 (0.974) |
|  | *M20 | 0.167 | 0.991 (0.974) |
|  | *M73 | 0.333 | 0.433 (0.35) |
|  | *M36 | 0.167 | 0.991 (0.89) |

(d) *Chaetoceros* sp. Na11C3

| **Dominant haplotype** | **Minor haplotype** | **D value** | **p value** |
| --- | --- | --- | --- |
| DM697 | *DM1718 | 0.333 | 0.433 (0.496) |
|  | *DM788 | 0.333 | 0.433 (0.512) |
|  | *DM622 | 0.333 | 0.433 (0.493) |
|  | *DM881 | 0.417 | 0.186 (0.196) |
|  | *DM907 | 0.333 | 0.431 (0.495) |
|  | #DM1213 | 0.583 | 0.019 (0.03) |

(e) *Chaetoceros* sp. Na26B1

| **Dominant haplotype** | **Minor haplotype** | **D value** | **p value** |
| --- | --- | --- | --- |
| DM638 | *DM1717 | 0.167 | 0.991 (0.854) |
|  | *DM1008 | 0.083 | 1 (1) |
|  | *DM706 | 0.083 | 1 (1) |
|  | *DM2633 | 0.083 | 1 (1) |
|  | #DM2555 | 0.083 | 1 (1) |

(f) *C. tenuissimus*

| **Dominant haplotype** | **Minor haplotype** | **D value** | **p value** |
| --- | --- | --- | --- |
| TM932 | *TM956 | 0.167 | 0.991 (0.979) |
|  | *TM926 | 0.167 | 0.991 (0.998) |
|  | *TM928 | 0.167 | 0.991 (0.999) |
|  | *TM942 | 0.167 | 0.991 (0.984) |
|  | *TM1131 | 0.25 | 0.786 (0.87) |
|  | #TM884 | 0.25 | 0.786 (0.849) |
|  | #TM1640 | 0.167 | 0.991 (0.997) |
|  | #TM920 | 0.5 | 0.066 (0.094) |
|  | #TM1445 | 0.5 | 0.066 (0.093) |
